# Polymer Network-Based Nanogels and Microgels: Design, Classification, Synthesis, and Applications in Drug Delivery

**DOI:** 10.3390/gels11090761

**Published:** 2025-09-22

**Authors:** Sabuj Chandra Sutradhar, Nipa Banik, Gazi A. K. M. Rafiqul Bari, Jae-Ho Jeong

**Affiliations:** 1 Department of Energy Materials Science and Engineering, Konkuk University, 268 Chungwon-aero, Chungju-si 27478, Republic of Korea; 2 Department of Mechanical Engineering, Gachon University, 1342 Seongnam-daero, Sujeong-gu, Seongnam-si 13120, Republic of Korea; 3 School of Mechanical Engineering, Chung-Ang University, 84 Heukseok-ro, Dongjak-gu, Seoul 06974, Republic of Korea

**Keywords:** nanogels, microgels, stimuli-responsive polymers, drug delivery, theranostics, personalized medicine

## Abstract

Polymer network-based nanogels (NGs) and microgels (MGs) have emerged as highly versatile platforms for advanced drug delivery, owing to their tunable architecture, biocompatibility, and responsiveness to diverse stimuli. This review presents a comprehensive and structured analysis of NG/MGs, encompassing their classification based on polymer origin, crosslinking mechanisms, composition, charge, stimuli-responsiveness, and structural architecture. We detail synthesis strategies—including inverse microemulsion and radiation-induced polymerization—and highlight key characterization techniques essential for evaluating physicochemical and functional properties. Emphasis is placed on the design-driven applications of NG/MGs in overcoming biological barriers and enabling targeted therapies, particularly in cancer, inflammation, diabetes, and viral infections. Multifunctional NGs integrating therapeutic and diagnostic capabilities (theranostics), as well as emerging platforms for immunotherapy and personalized medicine, are critically discussed. Finally, we address translational challenges and future directions, including scalable manufacturing, regulatory considerations, and integration with smart diagnostics. This review aims to serve as a foundational resource for researchers and clinicians developing next-generation NG/MG-based therapeutics.

## 1. Introduction

The development of advanced drug delivery systems has become a cornerstone of modern therapeutics, aiming to enhance efficacy, reduce side effects, and enable targeted treatment. Among these systems, polymer-based NG/MGs have garnered significant attention due to their unique structural and functional properties. These soft, water-swolled for accurate correspondence, crosslinked polymeric networks—ranging from nanometer to micrometer scales—offer high drug loading capacity, tunable release kinetics, and responsiveness to environmental stimuli [1].

NGs, typically sized between 1 and 1000 nm, and MGs, which span the micrometer range, are synthesized using natural or synthetic polymers. Their internal network structure allows them to absorb large amounts of water or biological fluids without dissolving, maintaining mechanical integrity, and enabling controlled drug release [2].

Natural polymers such as chitosan [3], alginate [4,5], gelatin [6], and hyaluronic acid [7] provide excellent biocompatibility and biodegradability, while synthetic polymers like poly(N-isopropylacrylamide) (PNIPAM) [8] and polyethylene glycol (PEG) [9] offer greater control over physicochemical properties and functionalization. MGs, ranging from hundreds of nanometers to several micrometers, are better suited for localized therapies [10], tissue engineering [11], and cell encapsulation [12,13]. Both systems are built upon polymer networks whose architecture—linear, branched, or dendritic—critically influences their mechanical strength, drug loading capacity, and diffusion dynamics.

A key advantage of NG/MGs lies in their stimuli-responsive behavior [14]. These systems can undergo reversible changes in response to physical (e.g., temperature, light, magnetic field), chemical (e.g., pH, redox conditions), or biological (e.g., enzymes, biomolecules) stimuli [15]. This responsiveness enables site-specific and controlled drug release, minimizing systemic toxicity and enhancing therapeutic efficacy [16]. For example, temperature-sensitive NGs based on PNIPAM can release drugs in response to hyperthermia, while pH-sensitive gels can target acidic tumor microenvironments or inflamed tissues [8].

Recent advances have focused on engineering NG/MGs with stimuli-responsive properties (e.g., pH, temperature, redox, and enzymatic), enabling on-demand drug release and real-time biosensing [17,18]. Functionalization with targeting ligands such as folate, RGD (Arginine-Glycine-Aspartic acid) peptides [19], and transferrin has further enhanced their specificity, allowing for tumor targeting, blood-brain barrier (BBB) penetration [20], and site-specific therapy [21]. In drug delivery, NGs have demonstrated the ability to encapsulate a wide range of therapeutic agents—including anticancer drugs [22], anti-inflammatory compounds [23,24], proteins [25], and nucleic acids [26]—and release them in response to physiological cues. Controlled release profiles ranging from hours to days have been achieved through careful tuning of crosslinking density, network porosity, and polymer degradation rates [27]. MGs, on the other hand, have shown promise in tissue engineering and regenerative medicine, where their modular structure, injectability, and mechanical adaptability support cell delivery, scaffold formation, and wound healing [12]. Their ability to mimic the extracellular matrix and promote cell infiltration has opened new avenues for 3D bioprinting and implantable therapies. In vivo studies and early-phase clinical trials have confirmed the enhanced bioavailability, reduced side effects, and improved patient compliance associated with NGs-based therapies. Applications span across oncology [27], neurology [28], diabetes [29], and infectious diseases [30], with several formulations advancing toward clinical translation [31].

Looking ahead, emerging trends include the development of hybrid NGs combining organic polymers with inorganic components (e.g., gold, silica, iron oxide) for theranostics, photothermal therapy, and magnetic targeting [32]. The integration of AI-guided design, microfluidic synthesis, and personalized medicine platforms is expected to accelerate the deployment of smart NGs tailored to individual patient needs [33].

Despite these advancements, several challenges remain in the clinical translation of NG/MG technologies. Issues such as scalability, reproducibility, long-term stability, and regulatory approval must be addressed to realize their full potential. Emerging trends in green chemistry and sustainable polymer design are paving the way for eco-friendly synthesis methods, while advances in manufacturing technologies are improving scalability and consistency [34].

This review is particularly timely and significant as it addresses the growing demand for multifunctional and stimuli-responsive drug delivery platforms capable of overcoming biological barriers and achieving site-specific therapeutic action. While several reviews have discussed NG/MGs, our manuscript provides a uniquely structured classification framework, a comparative analysis of synthesis techniques, and a broad overview of biomedical applications, including cancer therapy, inflammation control, and antiviral treatments. We also highlight emerging trends such as hybrid NGs, theranostic systems, and personalized medicine, which are reshaping the future of precision therapeutics.

By integrating recent advances in polymer chemistry, nanotechnology, and biomedical engineering, this review aims to serve as a comprehensive resource for researchers and clinicians seeking to understand and apply polymer network-based NG/MGs in next-generation drug delivery systems.

## 2. Classification of Polymeric NG/MGs

**Figure 1:**
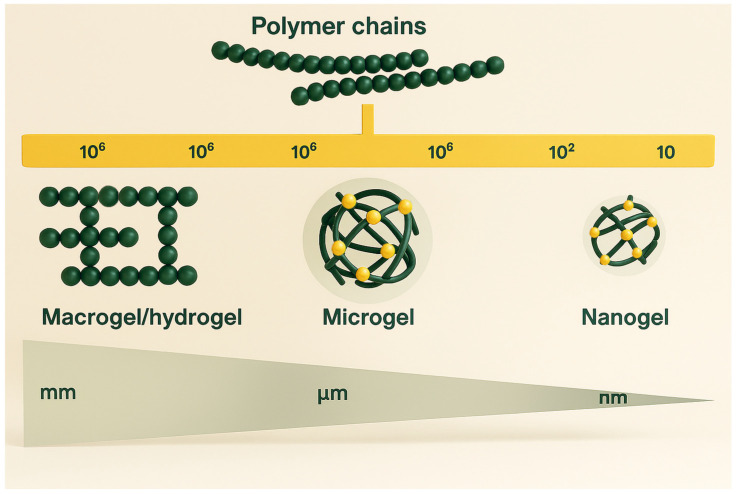
Illustration depicting gel structures across multiple size scales. Redrawn and adapted with permission from Ref. [36], Elsevier 2025.

Hydrogels are polymer networks formed by the combination of a dispersed phase (polymer chains) and a continuous phase (typically water). Depending on their particle size, they are categorized into macrogels, microgels, and nanogels. Macrogels are large, crosslinked structures that typically have a jelly-like consistency (Figure 1) [35]. In contrast, MGs and NGs are soft, smaller-scale materials formed by crosslinking polymers or biomolecules like proteins, which can create gel-like structures at microscopic or nanoscopic levels.

MGs generally range from 0.1 to 100 µm in diameter and are uniformly dispersed in a solvent where they swell. NGs are even smaller, typically sized between 10 and 100 nm. Unlike bulk hydrogels, MGs and NGs have a higher surface area exposed to the surrounding medium, allowing them to respond quickly to environmental stimuli such as pH, temperature, or ionic strength. This responsiveness enables them to expand or contract, a feature that solid particles lack due to their rigidity and limited interaction with solvents [37].

While NGs are often spherical, their shape can vary depending on the synthesis method and polymer architecture. For example, star-shaped NGs can be formed from polymers with multiple arms connected to a central core [38]. It’s also important to distinguish NG/MGs from polymeric micelles and surfactants. Unlike micelles, which are dynamic and undergo rapid monomer exchange, NG/MGs maintain a stable structure over time, making them more suitable for long-term applications. These properties make MGs and NGs particularly attractive for biomedical applications, including drug delivery, biosensing, and tissue engineering. Their ability to undergo reversible volume changes in response to stimuli allows for controlled release of therapeutic agents and dynamic interaction with biological environments.

**Scheme 1:**
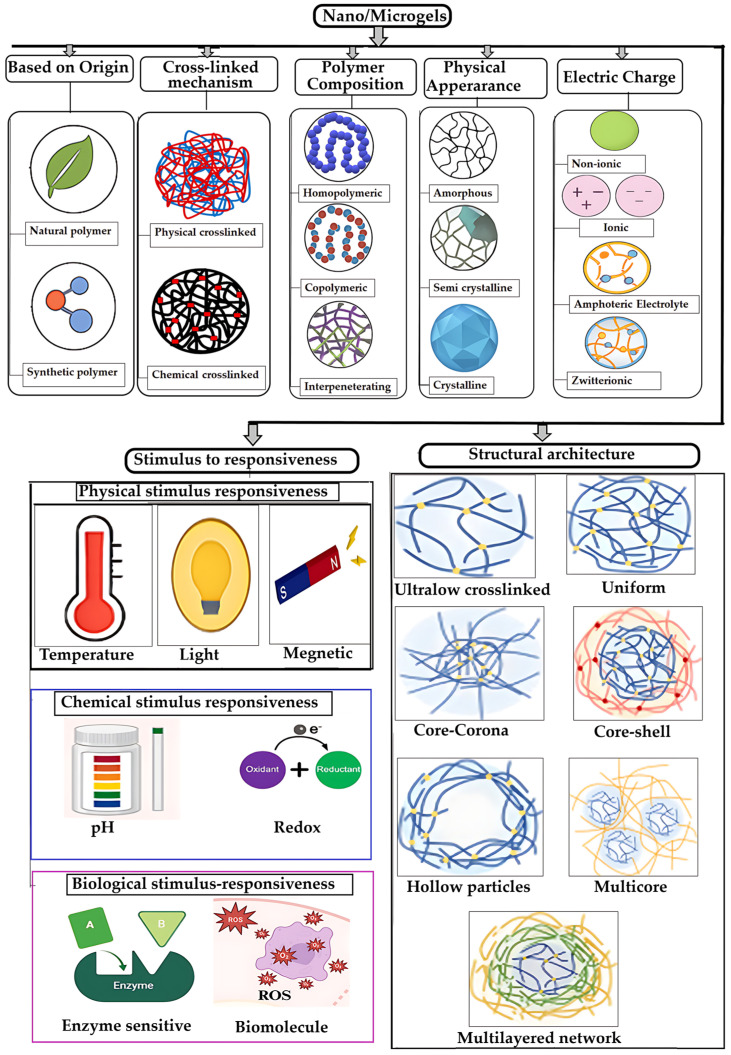
Schematic representation of various classes of polymeric NG/MGs. Redrawn and adapted with permission from [25], Elsevier 2025; [36,40], MDPI 2021.

Their versatility in biomedical applications—ranging from drug delivery to tissue engineering—is largely attributed to their tunable physicochemical properties. A comprehensive classification framework is essential for understanding their design, functionality, and therapeutic potential [39]. Polymer micro/NGs can be categorized based on several key criteria (Scheme 1).

### 2.1. Based on Origin of Polymers

The origin of the polymers used in NG/MGs synthesis plays a critical role in determining their physicochemical properties, biocompatibility, and suitability for biomedical applications. These polymers are broadly classified into natural, synthetic, and hybrid sources, each offering distinct advantages and limitations [41,42].

#### 2.1.1. Natural and Hybrid Polymer NG/MGs

Natural polymer gels are made from biopolymers commonly found in living organisms, such as polysaccharides (e.g., xanthan gum, alginate, starch, chitosan), proteins (e.g., fibrin, collagen, gelatin), and polynucleotides [43,44,45,46]. These materials are especially attractive due to their biodegradability and biocompatibility, making them safer and more environmentally friendly than many synthetic alternatives. Their natural origin and functional diversity have led to growing interest in biomedical and environmental applications. To create gels with tailored structures and properties, researchers often combine gelators of different molecular weights, resulting in supramolecular hybrid hydrogels [45]. These systems can exhibit unique mechanical and functional characteristics. When natural and synthetic polymers are blended, they form hybrid hydrogels. These materials combine the advantages of both components—such as the biodegradability of natural polymers and the mechanical strength and tunability of synthetic ones. This synergy allows for better control over properties like rigidity, viscosity, and durability [47,48].

#### 2.1.2. Synthetic Polymer NG/MGs

Synthetic polymer gels are engineered materials known for their tunable mechanical properties and controlled degradation behavior, making them suitable for a wide range of applications in biomedicine, materials science, and engineering [49,50,51]. These gels are commonly used in areas such as regenerative medicine, bioprinting, drug delivery, and energy storage. Some widely used synthetic polymers for gel formation include: Poly(ethylene glycol) (PEG) [52], Poly(vinyl alcohol) (PVA) [53], Poly(methyl methacrylate) (PMMA), Poly(hydroxyethyl methacrylate) (PHEMA) [54,55], Polyurethanes, Poly(amino acids), Poly(vinyl pyrrolidone) (PVP) [53]. These synthetic gels are often designed to mimic natural biopolymers, offering customizable features for specific applications. Many are developed as stimuli-responsive or environmentally sensitive polymers, capable of reacting to subtle changes in their surroundings. This responsiveness can manifest in various ways, such as: Sol-gel transitions, Changes in solubility, Alterations in shape or surface properties, Formation of complex gel structures [56,57]. Such dynamic behavior allows synthetic gels to adapt quickly and reversibly, making them ideal for advanced functional systems.

### 2.2. Based on Crosslinking Mechanism

The crosslinking mechanism is a fundamental criterion in the classification of polymeric NG/MGs, as it directly influences their structural integrity, responsiveness to environmental stimuli, and suitability for various biomedical applications. Crosslinking refers to the process by which polymer chains are interconnected to form a three-dimensional network, and it can occur through either physical or chemical interactions [58]. The nature of these interactions determines key properties such as mechanical strength, swelling behavior, degradation rate, and drug release kinetics [59]. Understanding the differences between physically and chemically crosslinked systems is essential for designing NGs tailored to specific therapeutic needs, whether for transient, reversible applications or long-term, stable drug delivery platforms.

#### 2.2.1. Physically Crosslinked NGs

Physically crosslinked NGs are formed through non-covalent interactions such as hydrogen bonding, ionic interactions, hydrophobic forces, and van der Waals associations, which result in reversible and stimuli-responsive networks. These gels are advantageous due to their ease of synthesis, as they do not require chemical crosslinkers, thereby minimizing residual toxicity. Their dynamic nature allows them to respond to environmental changes like pH, temperature, or solvent composition, making them ideal for applications in drug delivery, diagnostics, and biomedical devices [17,18,60,61,62,63,64]. Physical gels can be fabricated in various forms, including hydrogels, cryogels, aerogels, xerogels, films, and composites, and their behavior is influenced by polymer size and dispersion medium.

#### 2.2.2. Chemically Crosslinked NGs

In contrast, chemically crosslinked NGs are formed through covalent bonding between polymer chains, resulting in permanent, robust networks with controlled degradation and long-term stability. These gels are typically synthesized via methods such as addition or condensation polymerization, photopolymerization, and radiation-induced crosslinking, which can occur under mild conditions without added crosslinkers [65]. While chemical gels offer superior mechanical strength and are suitable for sustained release and implantable systems, they may involve more complex synthesis and potential limitations in hydrophilicity, especially when formed via ionic polymerization [17,66,67,68,69,70]. The choice between physical and chemical crosslinking significantly affects the gel’s swelling behavior, elasticity, and transport properties, and ultimately determines its suitability for specific biomedical or industrial applications.

### 2.3. Based on Polymeric Composition

Micro/NGs can be synthesized using various polymeric strategies, each offering distinct structural and functional attributes that influence their mechanical properties, responsiveness to stimuli, and compatibility with biological systems. These strategies are broadly categorized into homopolymeric, copolymeric, and interpenetrating polymer networks (IPNs), each with unique advantages for biomedical applications. Micro/NGs can be synthesized using various polymeric strategies, each offering distinct structural and functional attributes that influence their mechanical properties, responsiveness to stimuli, and compatibility with biological systems. These strategies are broadly categorized into homopolymeric, copolymeric, and interpenetrating polymer networks (IPNs), each with unique advantages for biomedical applications.

#### 2.3.1. Homopolymeric NG/MGs

Homopolymeric micro- and NGs are made from a single type of monomer. Depending on how the monomers are arranged during polymerization, the resulting gel networks can vary in structure and properties. For example, block sequences group monomers into distinct segments, which may lead to phase separation within the gel. Alternating sequences arrange monomers in a regular pattern, influencing mechanical strength and swelling behavior. Random sequences, on the other hand, distribute monomers without a fixed order, resulting in more heterogeneous networks. These gels are relatively simple in design and are often chosen when uniform chemical functionality is needed, such as in basic drug delivery systems or biosensors [70].

#### 2.3.2. Copolymeric NG/MGs

Copolymers are composed of two or more different monomers, typically including at least one hydrophilic component. This combination allows researchers to fine-tune the physical and chemical properties of the gel. By adjusting the ratio and type of monomers, scientists can control the gel’s hydrophilicity, mechanical strength, degradation rate, and responsiveness to stimuli like pH or temperature. Copolymeric NGs are especially useful in biomedical applications because they offer greater flexibility in design. They can be engineered to carry specific drugs, respond to environmental changes, or interact with biological targets, making them ideal for targeted and controlled drug delivery [15,16,71,72].

#### 2.3.3. Multipolymer Interpenetrating Polymer Networks (IPNs)

Interpenetrating polymer networks (IPNs) are more complex structures formed by combining two or more polymer networks. Typically, one network is chemically crosslinked, while the other remains linear or loosely associated. These networks are physically intertwined but not covalently bonded. IPNs can be made from synthetic, natural, or hybrid polymers, and their unique architecture provides enhanced mechanical strength and multifunctionality. The combination of different polymers allows IPNs to respond more effectively to environmental stimuli such as changes in pH or temperature. Because of their structural complexity and adaptability, IPNs are widely used in tissue engineering and advanced drug delivery systems [11,73,74].

### 2.4. Based on Physical Appearance

The internal structure of micro- and NGs plays a key role in how they behave in solution and interact with biological systems. Based on their physical appearance, these gels can be categorized as amorphous, semicrystalline, or crystalline [75]. Amorphous gels lack a defined structure, making them flexible and highly responsive to stimuli. They often swell significantly when exposed to water or biological fluids. Semicrystalline gels contain both ordered and disordered regions, offering a balance between mechanical strength and flexibility. Crystalline gels have a highly ordered structure throughout, which gives them rigidity and stability but may limit their swelling and responsiveness. The choice of gel type depends on the intended application and desired performance characteristics.

### 2.5. Based on Electrical Charge of the Network

The electrical charge of a gel’s polymer network significantly affects its interaction with biological molecules, membranes, and external stimuli. Micro- and NGs can be neutral, ionic, amphoteric, or zwitterionic [75]. Neutral gels do not carry a net charge and are often used in applications where minimal interaction with charged species is preferred. Ionic gels can be either anionic or cationic. Anionic gels contain negatively charged groups like carboxylates or sulfates, which can bind to positively charged drugs or proteins. Cationic gels have positively charged groups such as amines, making them suitable for delivering negatively charged biomolecules like DNA or RNA.

Amphoteric gels contain both acidic and basic functional groups, allowing them to respond dynamically to changes in pH. This makes them particularly useful in environments where pH fluctuates, such as inflamed tissues or tumor sites. Zwitterionic gels are unique in that each repeating unit contains both positive and negative charges, resulting in an overall neutral charge. These gels are highly biocompatible and resistant to protein adsorption, making them ideal for implantable devices and biosensors.

### 2.6. Based on Responsiveness to Stimuli

**Figure 2:**
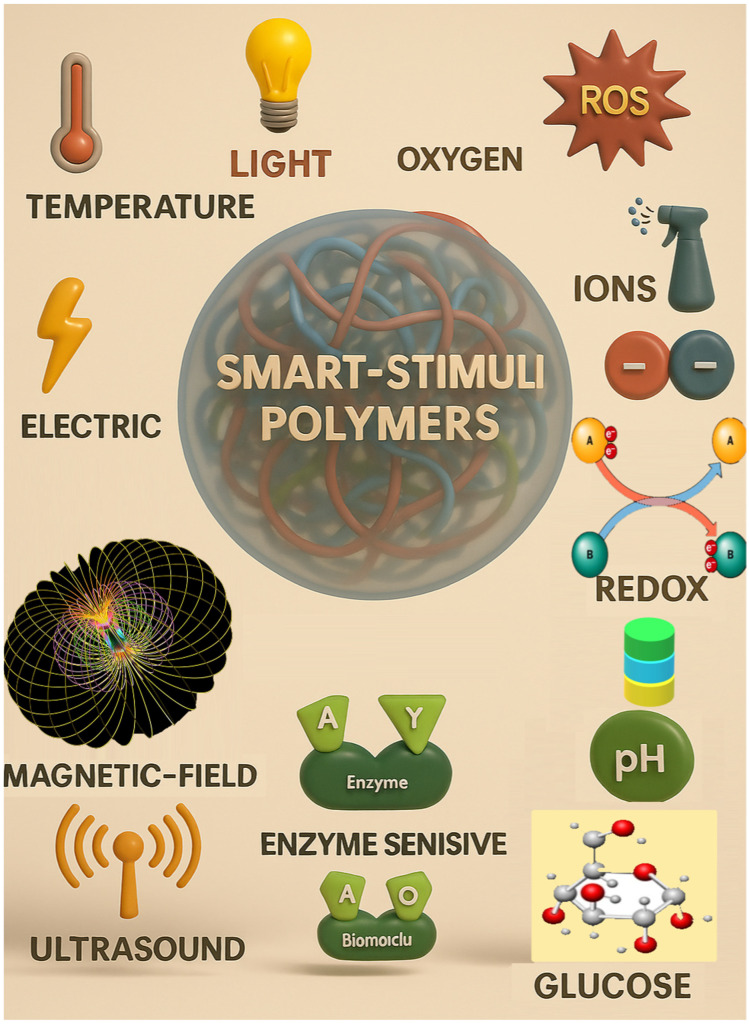
Schematic representation of polymeric MG/NGs classification based on stimulus-responsiveness. Reproduced with permission from Motornov et al. [78], MDPI 2024.

Stimulus-responsive micro/NGs are designed to remain stable under normal physiological conditions but undergo controlled changes in response to specific external triggers. This property is particularly valuable in drug delivery, where NGs must circulate intact in the bloodstream and release their payload only at targeted sites. These stimuli can be physical (e.g., temperature, light, ultrasound, magnetic or electric fields, pressure), chemical (e.g., pH, redox potential), or biological (e.g., enzymes, biomolecules) [76,77] (Figure 2). The response mechanism typically involves signal detection, internal structural or chemical transformation, and a visible or functional change in the NGs [78].

#### 2.6.1. Physical Stimulus-Responsiveness

NG/MGs can be engineered to respond to various physical stimuli, enabling precise control over drug release and therapeutic action. Temperature-responsive NGs exhibit a volume phase transition temperature (VPTT), where their size changes due to altered interactions among polymer chains and solvents. These NGs are classified based on their response direction: those with a lower critical solution temperature (LCST) shrink above a threshold, while those with an upper critical solution temperature (UCST) swell when heated. Materials like PNIPAM, PNVCL, and Pluronic copolymers are commonly used to engineer such systems [77,79,80,81,82,83,84,85,86,87]. For instance, Peng et al. developed a zwitterionic NGs (PMEDAPA-Tf@DOX) that responds to mild microwave-induced hyperthermia (~41 °C) for targeted tumor therapy [88]. Light-responsive NGs react to UV, visible, or near-infrared (NIR) light through physical changes (e.g., heat generation) or chemical transformations (e.g., bond cleavage, isomerization). These responses are often driven by functional groups in the polymer or embedded photoactive agents like metal nanoparticles [76,77,89]. Ultrasound-responsive NGs utilize mechanical or thermal effects from ultrasonic waves (20 kHz to >50 MHz) to enhance drug release and tissue penetration. Cavitation and localized heating facilitate these effects, even in hollow NGs without gas-filled microbubbles [89,90,91,92]. Magnetic field-responsive NGs incorporate magnetic nanoparticles to enable non-invasive control over drug release, imaging, and hyperthermia. Their behavior can be tuned by adjusting the field strength, making them suitable for real-time therapeutic applications [77,93,94,95]. Electro-responsive NGs contain polymers that react to electric fields through dipole orientation or redox reactions. Though less common, they hold promise for treating electrically active tissues such as nerves and muscles. For example, Ying et al. developed NGs that released antiepileptic drugs in response to abnormal brain electrical signals [96,97,98]. Lastly, pressure-responsive NGs are designed with mechano-sensitive components that deform under mechanical stress, enabling applications in cardiovascular and gastrointestinal therapies. These systems often rely on unstable complexes that undergo structural changes when compressed [99,100].

#### 2.6.2. Chemical Stimulus-Responsive Micro/NGs

Stimulus-responsive NGs can be engineered to react to specific chemical or biological signals, enabling controlled structural changes and functional responses. Among chemical triggers, pH-responsiveness is one of the most widely studied. NGs designed with pH-sensitive polymers respond to environmental pH shifts through mechanisms such as protonation/deprotonation or cleavage of acid-labile bonds like hydrazone, imine, oxime, acetal, and ketal [101]. These systems exhibit a volume-phase transition pH (VPTpH), where the gel swells below a threshold and shrinks above it [102]. This property is particularly useful in drug delivery, as different tissues and cellular compartments exhibit distinct pH levels—for example, blood (~7.4), tumor microenvironments (lower pH), and intracellular organelles like lysosomes (~4.5–5.0) [69,95]. Redox-responsive NGs leverage physiological redox gradients, such as the difference in glutathione (GSH) concentration between intracellular (2–10 mM) and extracellular (2–20 μM) environments, or between healthy and diseased tissues [77,103]. These NGs often incorporate disulfide bonds, which cleave under reducing conditions, enabling targeted drug release in tumor cells or inflamed tissues [104]. Glucose-responsive gels typically utilize boronic acid derivatives or glucose-sensitive enzymes that interact with glucose molecules, triggering changes in the gel structure [105]. CO_2_-responsive gels contain functional groups like amines or carbonate linkages that react with CO_2_, leading to reversible changes in gel properties [105,106]. Hypoxia-responsive gels are tailored to respond to low oxygen levels, often found in tumor microenvironments. These gels release their payload selectively under hypoxic conditions, enhancing therapeutic specificity [7].

#### 2.6.3. Biological Stimulus-Responsive Micro/NGs

Biological stimuli play a crucial role in the design of smart micro/NGs, enabling them to respond selectively to specific biochemical signals present in pathological environments. These stimuli include enzymes, biomolecules (such as glucose, ATP, and reactive oxygen species), and cellular conditions that are often dysregulated in diseases like cancer, inflammation, and infections. By incorporating biologically sensitive moieties into their polymer networks, NGs can undergo structural or chemical transformations that trigger drug release, change surface properties, or activate therapeutic functions. Enzyme-sensitive NGs are designed with substrates or analogues that can be cleaved by specific enzymes, leading to structural changes and functional activation. Enzymes such as matrix metalloproteinases (MMPs), trypsin, phosphatases, and elastases have been used to trigger responses in NGs for drug release or biosensing applications [107,108,109,110,111,112]. These systems are particularly valuable in disease contexts where enzyme expression is dysregulated. Additionally, NGs can respond to biomolecules like glucose, reactive oxygen species (ROS), and adenosine triphosphate (ATP) [113,114]. Glucose-responsive NGs can be constructed using glucose oxidase (GOx), lectins like concanavalin A (Con A), or phenylboronic acid (PBA) conjugation. These mechanisms enable glucose detection or insulin release in diabetic therapy [105,106]. ROS-responsive NGs incorporate functional groups such as diselenides, arylboronic esters, thioethers, and thioketals, which undergo bond cleavage or solubility changes upon exposure to ROS like H_2_O_2_, •OH, or ^1^O_2_ [115,116]. To enhance precision and versatility, many NGs are now designed to be dual- or multi-responsive, integrating multiple triggers such as pH and redox or temperature and light. These advanced systems allow for spatiotemporal control over drug release and degradation, broadening their potential in targeted therapy, diagnostics, and smart biomaterials [117,118,119,120,121].

### 2.7. Based on Structural Architecture

The internal structure of gel particles is influenced by factors such as the choice of monomers, crosslinkers, and the kinetics of particle formation, which together determine the segment density and spatial organization within the gel. Even when composed of homopolymers, these variables can lead to diverse architectural configurations. Table 1 illustrates the versatility in achieving different architectural configurations.

## 3. Synthesis and Characterization of Micro/NGs

### 3.1. Synthesis Methods

MGs and NGs are synthesized through either chemical or physical crosslinking of hydrophilic polymers, each offering distinct structural and functional properties. Physical crosslinking relies on reversible, non-covalent interactions such as ionic, hydrophobic, and hydrogen bonding forces, enabling responsiveness to environmental stimuli and biodegradability—features particularly valuable for drug, protein, or cell encapsulation and release. In contrast, chemically crosslinked gels are formed via covalent bonds, providing enhanced structural stability and typically requiring crosslinking agents [131,132,133].

Effective synthesis of micro/NGs demands precise control over particle size distribution, functional group placement, and colloidal stability [131,132]. MG fabrication methods are broadly categorized into homogeneous nucleation and polymerization, emulsification, and polymer complexation. Homogeneous nucleation involves mixing water-soluble monomers, crosslinkers, and initiators in aqueous media, often via emulsion polymerization; sequential polymerization can yield core-shell structures. Emulsification techniques disperse aqueous polymer droplets in oil, followed by crosslinking using difunctional or multifunctional agents. Other methods include precipitation, inverse (mini) emulsion, inverse microemulsion, dispersion polymerization, membrane emulsification, and controlled/living radical polymerization [131,132]. Polymer complexation involves mixing oppositely charged polyelectrolytes or hydrogen-bonding polymers to form physically crosslinked networks, with factors such as hydrophobicity, molecular weight, polymer structure, solvent, and pH influencing gel formation and stability [134,135].

NGs follow similar principles but are often tailored for biomedical applications, where dispersion stability is critical. Their synthesis is generally categorized into crosslinking polymerization—using monomers and crosslinkers—and crosslinking of polymer precursors, such as amphiphilic or reactive polymers capable of self-assembly. Additional techniques include physical self-assembly in aqueous media, forming NGs through hydrogen bonding, van der Waals forces, and electrostatic interactions, and template-assisted fabrication using photolithography or micromolding, which enables precise particle design [136,137,138,139,140].

A particularly clean and efficient method involves ionizing radiation, such as gamma rays or fast electron beams, which initiate intramolecular crosslinking in water-soluble polymers without the need for monomers, surfactants, or crosslinking agents—thus eliminating purification steps [136,141]. This technique allows fine control over NGs’ properties and supports biomedical applications. However, its scalability is limited due to the complexity and cost of high-energy radiation equipment [136,141,142,143]. The synthesis methods of micro- and NGs are summarized in Table 2.

### 3.2. Comparative Evaluation of NG/MGs Synthesis Techniques

NG/MGs synthesis methods vary widely in their mechanisms, scalability, and compatibility with different polymer types. Among these, inverse microemulsion polymerization and radiation-induced polymerization are particularly noteworthy due to their unique advantages and limitations.

#### 3.2.1. NGs Fabrication by Inverse Microemulsion

Inverse microemulsion polymerization confines free-radical polymerization within nanometer-sized aqueous droplets dispersed in a continuous oil phase, stabilized by surfactants and co-surfactants. These droplets act as nanoreactors, and their size—and thus NG diameter—can be tuned by adjusting the water-to-surfactant ratio (W_0_), surfactant hydrophilic-lipophilic balance (HLB), oil composition, and ionic strength. Water-soluble monomers such as acrylamide, N-isopropylacrylamide (NIPAM), and PEG derivatives, along with initiators, are localized in the dispersed phase. Polymerization is typically initiated thermally or via redox systems, yielding crosslinked NGs with narrow size distributions [154,155,156,157,158]. This method generally produces NGs in the range of 20–200 nm with low polydispersity, high encapsulation efficiency for hydrophilic payloads, and the ability to incorporate stimuli-responsive functionalities (e.g., pH or temperature sensitivity using NIPAM, MAA, or AMPSA) [156,157]. Recent studies have demonstrated tunable responsiveness by varying comonomer ratios and dispersed-phase volume, while environmentally friendly formulations using optimized HLB surfactants and lower processing temperatures have also been reported [156]. Classical kinetic studies of inverse miniemulsions provide further insight into rate behavior and stabilization mechanisms [155]. As a representative example, Peters et al. synthesized core–shell PNIPAM-based NGs for controlled chemotherapeutic release, achieving precise particle size and drug-loading control, though requiring prolonged dialysis to remove residual surfactants [8]. The main advantages of this technique include (i) precise size control and uniformity via droplet nanoreactors, (ii) high encapsulation efficiency for hydrophilic drugs, proteins, and nucleic acids, and (iii) mild processing conditions suitable for temperature-sensitive biomolecules [157,158].

However, limitations remain, such as (i) surfactant removal challenges requiring extensive dialysis or ultrafiltration, (ii) scale-up limitations due to microemulsion stability and phase separation, and (iii) restricted monomer scope favoring water-soluble vinyl monomers [155,156].

#### 3.2.2. Radiation-Induced Polymerization

Radiation-induced polymerization utilizes ionizing radiation sources, including gamma rays, electron beams, or X-rays, to initiate polymerization and crosslinking processes without the need for chemical initiators. Radiolysis of water generates reactive species (e.g., hydroxyl radicals, hydrated electrons) that initiate and propagate polymer chains. The absorbed dose and dose rate determine crosslink density, NG size, and swelling behavior [159]. Because radiation simultaneously sterilizes the material, this method is highly attractive for producing clinical-grade NGs and supports continuous processing using conveyor-based electron-beam systems [160,161]. Radiation-based methods have been successfully applied to synthesize clean, injectable NGs from polymers such as PVA, PEG, PVP, chitosan, and poly(acrylic acid). Incorporation of comonomers like NIPAM or DMAEMA enables stimuli-responsive behavior [160,162]. Recent studies have reported pH-responsive pectin/PAA NGs for cancer therapy and injectable hydrogels for wound healing and tissue engineering [12,162]. As a representative example, Basak et al. demonstrated the fabrication of enzyme-responsive PEG NGs via gamma irradiation in confined aqueous droplets, achieving simultaneous crosslinking and sterilization for cardiovascular applications [9]. The main advantages of this approach include (i) initiator- and crosslinker-free synthesis, minimizing residual toxicity and enhancing biocompatibility, (ii) simultaneous sterilization, eliminating additional processing steps, and (iii) scalable, continuous manufacturing, particularly with electron-beam systems [12,161].

However, limitations include (i) dependence on specialized facilities for gamma or electron-beam irradiation, (ii) payload radiosensitivity, which may lead to degradation of proteins or nucleic acids, and (iii) polymer specificity, favoring radiation-sensitive backbones or vinyl-functionalized polymers [12,161].

#### 3.2.3. Comparative Perspective and Design Guidance

Inverse microemulsion polymerization is preferred when precise size control (20–200 nm) and high loading of hydrophilic therapeutics are critical, provided that surfactant removal and batch processing are acceptable [157,158]. Radiation-induced polymerization is ideal when ultra-clean, initiator-free NGs and in-line sterilization are required, or when continuous electron-beam processing is available—assuming the payload can tolerate radiation exposure [12,161]. Table 3 summarizes the inverse microemulsion and radiation-induced NG/MGs fabrication routes.

#### 3.2.4. Practical Parameter Windows

To achieve reproducible and application-specific NG properties, both inverse microemulsion polymerization and radiation-induced polymerization require careful control of key synthesis parameters. These parameters influence droplet stability, crosslinking behavior, responsiveness, and ultimately the scalability and functionality of the resulting NGs.

In inverse microemulsion polymerization, critical factors include the water-to-surfactant ratio (W_0_), the hydrophilic-lipophilic balance (HLB) of the selected surfactants, and the composition of the oil phase. Optimizing these variables ensures stable droplet formation and minimizes phenomena such as Ostwald ripening, which can compromise NG uniformity. Responsiveness to environmental stimuli can be finely tuned by incorporating comonomers such as N-isopropylacrylamide (NIPAM) with methacrylic acid (MAA) or 2-acrylamido-2-methylpropane sulfonic acid (AMPSA), enabling dual pH- and temperature-sensitive behavior [156,157].

In radiation-induced polymerization, the absorbed dose (kGy) and dose rate are key determinants of crosslink density and NG size. Maintaining the polymer concentration within the dilute-to-semi-dilute regime favors intramolecular crosslinking, which is essential for forming discrete NGs and avoiding undesirable bulk gelation [160,161]. This approach is particularly advantageous for synthesizing sterile, initiator-free NGs suitable for biomedical use.

A comparative overview of the scalability and application relevance of these two techniques is presented in Table 4, highlighting their respective strengths and limitations across research and industrial contexts.

#### 3.2.5. Strategic Selection Based on Application

The choice between inverse microemulsion polymerization and radiation-induced polymerization depends on the intended application, processing constraints, and regulatory considerations. For precision drug delivery, where tight size control (20–200 nm) and high encapsulation efficiency of hydrophilic therapeutics are critical, inverse microemulsion polymerization is generally preferred [157,158]. This method allows fine-tuning of NG size and responsiveness (e.g., pH or temperature sensitivity) through control of the water-to-surfactant ratio (W_0_), surfactant HLB, and comonomer composition, making it highly suitable for academic research and preclinical studies where batch processing and extensive purification steps are acceptable [156,158]. However, its scalability is limited by the complexity of microemulsion stabilization and the need for surfactant removal, which can impact biocompatibility [156,160].

In contrast, radiation-induced polymerization offers a cleaner, initiator-free synthesis route with built-in sterilization, making it particularly attractive for clinical translation and large-scale manufacturing [162]. This approach eliminates toxic initiators and residual surfactants, thereby improving regulatory compliance and reducing downstream purification requirements. Furthermore, the ability to integrate continuous processing using electron-beam systems enhances industrial feasibility for producing injectable hydrogels, wound dressings, and implantable biomaterials [12,162]. Nevertheless, this method requires access to specialized irradiation facilities and careful consideration of payload radiosensitivity, as sensitive biomolecules (e.g., proteins, nucleic acids) may degrade under high-energy exposure [9].

Overall, inverse microemulsion polymerization is strategically suited for customized NG systems in research and early-stage development, while radiation-induced polymerization provides a regulatory-friendly, scalable pathway for clinical-grade products, particularly in applications demanding sterility and high purity, such as injectable drug delivery systems and tissue engineering scaffolds.

### 3.3. Tools and Techniques for Characterizing NG/MGs

**Figure 3:**
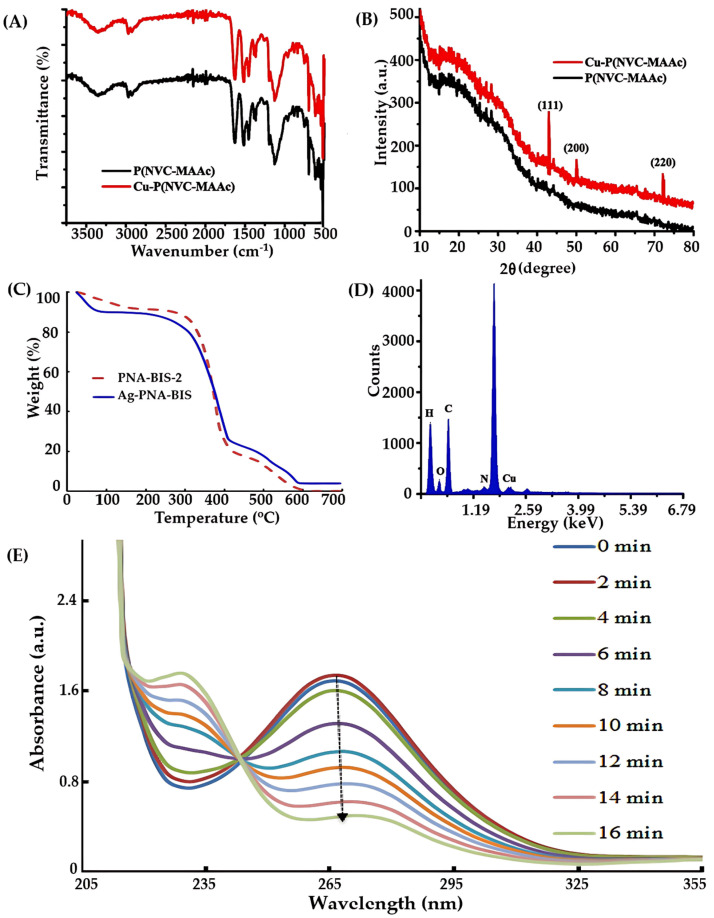
Characterization techniques for NGs: (**A**) FTIR [165], (**B**) XRD [165], (**C**) TGA [166], (**D**) EDX [165], and (**E**) UV/Vis spectra [165]. (**A**,**B**,**D**,**E**) adopted with permission from ref. [165], Elsevier 2019 and C adopted with permission from ref. [166], Elsevier 2019.

A wide array of analytical techniques is employed to investigate the size, structure, composition, behavior, and appearance of metal nanoparticle-based NG/MGs. These methods are essential for distinguishing NG/MGs from their corresponding smart NG/MGs and for understanding their physicochemical properties. Figure 3A–E illustrates representative examples of key characterization techniques used in this context.

#### 3.3.1. Chemical Functionalities

Fourier-transform infrared spectroscopy (FTIR), shown in Figure 3A, is commonly used to identify functional groups and assess chemical interactions within the polymer matrix. It provides insights into the bonding environment and the incorporation of metal nanoparticles into the gel network. Complementary techniques such as ^1^H-nuclear magnetic resonance (NMR) and Raman spectroscopy (RS) are also widely applied to investigate the molecular structure and functional modifications of the organic polymer components.

X-ray diffraction (XRD), depicted in Figure 3B, is used to determine the crystalline nature of both the polymer matrix and embedded metal nanoparticles. Energy-dispersive X-ray spectroscopy (EDX) complements XRD by confirming the elemental composition and distribution of metal species within the gel. These techniques are crucial for verifying the successful incorporation and stabilization of nanoparticles in hybrid systems.

Thermal stability and decomposition behavior are assessed using thermogravimetric analysis (TGA), differential scanning calorimetry (DSC), and differential mechanical analysis (DMA). TGA, shown in Figure 3C, is particularly useful for quantifying the metal content in hybrid MGs and evaluating their thermal degradation profiles. These thermal techniques help determine the suitability of hybrid MGs for temperature-sensitive applications.

Energy-dispersive spectroscopy (EDS), illustrated in Figure 3D, is employed to analyze the elemental composition of hybrid MGs. It provides detailed information about the distribution and concentration of metal nanoparticles within the gel matrix. Inductively coupled plasma techniques, including ICP-MS, ICP-OES, and ICP-AES, are also used to quantify the presence of noble metals and assess their uniformity across samples.

Optical techniques such as UV-Visible/NIR spectroscopy (Figure 3E) are used to monitor the volume phase transition temperature (VPTT) of MGs and hybrid systems by measuring turbidity. These methods also confirm the formation and stabilization of plasmonic nanoparticles within the gel network.

#### 3.3.2. Morphological Characterization

**Figure 4:**
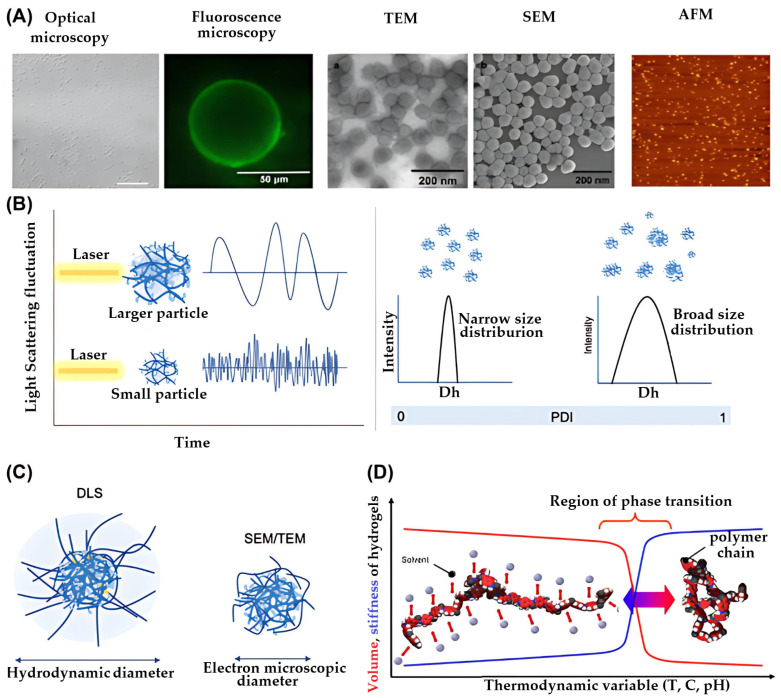
(**A**) Representative of particle morphology characterized by different microscopy [167,168,169], reproduced with permission from ref. [154]. Springer 2016; reproduced with permission from ref. [155]. Beilstein-Institut 2015; reproduced with permission from ref. [156]. Elsevier 2015. (**B**) Illustration of dynamic light scattering of larger particles and smaller particles (left) and DLS profile of particles with narrow size distribution and broad size distribution (right) (**C**), schematic illustration presenting the measurement of particle size from DLS, SEM, and TEM and (**D**) the illustration presenting the interaction between polymer-polymer and polymer-solvent under specific environment, reproduced with permission from ref. [36]. Elsevier 2025.

Scanning Electron Microscopy (SEM) and Transmission Electron Microscopy (TEM) are essential for visualizing particle morphology. SEM provides 3D-like surface images with high depth of field, useful for examining topography and aggregation. TEM offers high-resolution 2D images of internal structures and compartments. Figure 4A illustrates TEM imaging of NGs/MGs. When combined with Energy-Dispersive X-ray Spectroscopy (EDS), SEM can also provide elemental composition data.

Atomic Force Microscopy (AFM) is used to analyze surface roughness, particle height, and mechanical properties such as stiffness. It operates by scanning a sharp probe across the sample surface and measuring interaction forces. Phase imaging modes allow for the detection of chemical heterogeneity.

Fluorescence Microscopy enables visualization of the spatial distribution within NGs/MGs. It can distinguish layers or compartments using fluorescent dyes or intrinsic fluorescence, revealing complex internal structures.

#### 3.3.3. Particle Size and Size Distribution

Dynamic Light Scattering (DLS) is a non-invasive technique that measures particle size based on light scattering caused by Brownian motion. It provides the hydrodynamic diameter, which includes both the particle and its surrounding solvent shell. This value is typically larger than the size measured by SEM or TEM, which observes particles in a collapsed or dried state. Figure 4B (left) shows DLS data for particle size, while Figure 4C compares hydrodynamic and collapsed sizes.

DLS is also used to assess size distribution and detect aggregates. The polydispersity index (PDI) derived from DLS reflects the uniformity of particle sizes. A PDI below 0.5 indicates a narrow distribution, while values above 0.7 suggest significant heterogeneity, as shown in Figure 4B (right).

#### 3.3.4. Macroscopic Properties

Although NGs and MGs are nano-/microscale materials, they exhibit macroscopic behaviors that are observable and relevant to their practical applications. These include turbidity, swelling, viscosity, and rheological behavior.

##### Turbidity and Swelling Behavior

**Figure 5:**
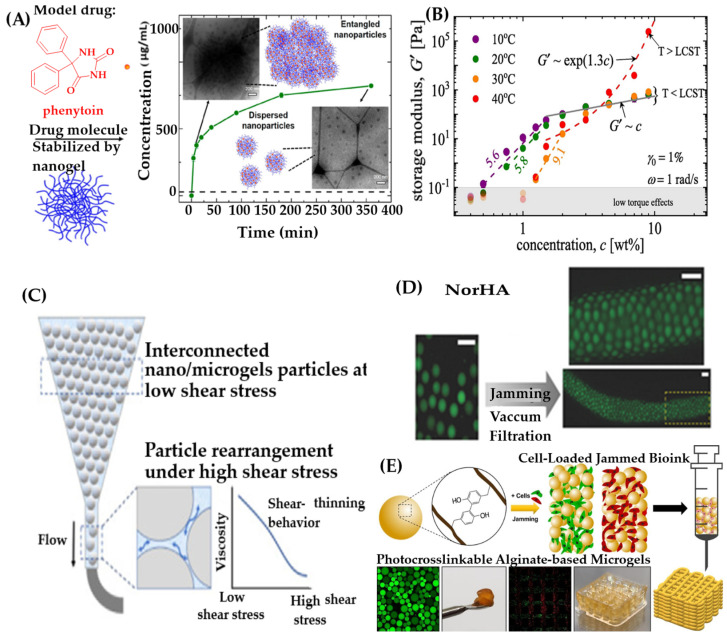
(**A**) Effect of concentration on particle aggregation, reproduced with permission from ref. [170]. ACS 2019; (**B**) Concentration-dependent elastic modulus at different temperatures, reproduced with permission from ref. [171]. Elsevier 2021; (**C**) Shear-thinning behavior of gel-based inks for 3D printing, reproduced with permission from ref. [36]. Elsevier 2025; (**D**) Fluorescence images of extruded MGs, reproduced with permission from ref. [172]. Wiley 2019; (**E**) Schematic of cell-laden granular MG bioink for 3D bioprinting, reproduced with permission from ref. [173]. Elsevier 2023.

The swelling behavior of NGs/MGs is governed by interactions among polymer chains, solvent molecules, and ionic species. Figure 4D illustrates phase separation driven by polymer–polymer interactions, which promote shrinkage, and polymer–solvent interactions, which promote swelling. Temperature plays a critical role in modulating these interactions. At lower temperatures, hydrogen bonding between polymer and solvent dominates, leading to swelling. Above the lower critical solution temperature (LCST), hydrophobic interactions prevail, causing shrinkage. UV-Vis spectroscopy is used to measure turbidity by analyzing light transmittance and absorbance. DLS also helps monitor aggregation and packing density in solution. As shown in Figure 5A, increased concentration leads to tighter packing and reduced light transmission.

##### Rheological Properties

Rheological behavior reflects how NGs/MGs respond to mechanical stress. At low concentrations, particles are dispersed and exhibit viscous behavior. As concentration increases, physical interactions intensify, leading to viscoelastic behavior—a combination of fluid-like and solid-like properties. This transition is marked by increases in viscosity and modulus, indicating greater stiffness and resistance to deformation. Figure 5B shows that PNIPAM-based MGs exhibit a sharp increase in storage modulus above their LCST (~40 °C), especially at higher concentrations. This is due to attractive interactions and physical bonding among compact PNIPAM globules. Rheological responses such as shear-thinning and shear-thickening are critical for applications like injectable hydrogels and 3D printing. Shear-thinning facilitates smooth extrusion during printing, while shear-thickening increases resistance to flow under stress. In extrusion-based 3D printing, NG/MG-based bio-inks offer advantages over macrogels. Figure 5C illustrates shear-thinning behavior that enables precise deposition. After extrusion, gels can solidify via self-assembly or external stimuli such as temperature or light. Moreover, NG/MG-based inks address limitations of macrogels, such as restricted cell migration and nutrient exchange. The porous structure formed by loosely packed NGs/MGs facilitates efficient transport of oxygen, nutrients, and waste, supporting cell viability and function within printed scaffolds (Figure 5D,E).

### 3.4. Limitations of Common NG/MGs Characterization Techniques

Accurate characterization of NG/MGs is essential for understanding their physicochemical properties, optimizing formulation strategies, and ensuring reproducibility in biomedical applications. A wide array of analytical techniques is employed to assess particle size, morphology, chemical composition, and mechanical behavior. However, each method has inherent limitations that must be critically considered to avoid misinterpretation of data.

Dynamic Light Scattering (DLS) is one of the most commonly used techniques for estimating the hydrodynamic diameter of particles based on Brownian motion. While DLS is rapid and non-invasive, it is highly sensitive to the presence of aggregates. Even minor aggregation can lead to significant overestimation of particle size and polydispersity index (PDI), potentially misrepresenting the true size distribution of the sample [58,169]. Moreover, DLS cannot differentiate between single particles and loosely bound clusters, making it less reliable for heterogeneous or polydisperse systems [174]. For example, Fissan et al. [175] demonstrated that DLS failed to resolve binary nanoparticle mixtures, whereas SEM and analytical disc centrifugation (ADC) provided clear differentiation.

Scanning Electron Microscopy (SEM) and Transmission Electron Microscopy (TEM) offer high-resolution imaging of particle morphology and internal structure. However, these techniques require extensive sample preparation, including drying, staining, and exposure to vacuum conditions. Such steps can introduce artifacts like shrinkage, deformation, or aggregation, which may not reflect the native hydrated state of NGs [167,169]. Additionally, these methods provide only static snapshots and cannot capture dynamic behavior in physiological environments. The use of contrast agents or staining in TEM can further obscure fine structural details or interfere with interpretation.

To overcome these limitations, complementary techniques such as Atomic Force Microscopy (AFM), Cryo-TEM, and nanoparticle tracking analysis (NTA) are often employed. AFM enables imaging under ambient conditions without vacuum or staining, preserving hydrated morphology. Cryo-TEM allows visualization of NGs in their frozen hydrated state, minimizing structural distortion. Nevertheless, AFM is limited by its small scan area and slow imaging speed, making it less suitable for statistically representative sampling [176].

Other characterization methods also present specific challenges. Fourier Transform Infrared Spectroscopy (FTIR) is widely used to identify functional groups, but overlapping peaks and low sensitivity to minor chemical changes can hinder precise interpretation [58]. UV-Visible spectroscopy is useful for monitoring optical properties and phase transitions, yet sample turbidity and scattering can distort absorbance readings [175]. Thermogravimetric Analysis (TGA) provides insights into thermal stability and composition but requires dry samples and cannot capture behavior under physiological conditions [169]. Rheological measurements are essential for understanding viscoelastic properties, but results are influenced by concentration, temperature, and shear history, and may not directly correlate with in vivo performance [171].

In summary, no single technique can comprehensively characterize NG/MGs. A multi-technique strategy, combining complementary methods and careful experimental design, is crucial for obtaining accurate, reproducible, and physiologically relevant data.

## 4. Applications of Nano/Microgels (NG/MG) for Drug Delivery

### 4.1. Limitations, Translational Barriers, and Comparative Performance of Conventional Nanocarriers

Despite the growing interest in nanocarrier-based drug delivery systems, several translational barriers continue to hinder their clinical success [177]. Among the most widely studied nanocarriers—liposomes, dendrimers, and polymeric micelles—each presents unique advantages and limitations. Liposomes are clinically established and biocompatible, capable of encapsulating both hydrophilic and hydrophobic drugs. However, they often suffer from rapid clearance, limited long-term stability, and restricted drug loading efficiency, particularly for macromolecules like proteins. Although advanced methods such as base exchange (e.g., Doxil) have improved drug loading for certain compounds, their applicability remains narrow [178].

Dendrimers, with their hyperbranched architecture, offer precise molecular design and high drug-loading capacity. They can encapsulate small molecules and even nanoparticles [179,180,181]. Nevertheless, their complex synthesis, high cost, and potential cytotoxicity—especially due to high positive charge densities—limit their widespread use [182,183]. Cationic dendrimers and liposomes, while effective as nonviral gene delivery vectors, also raise concerns about immunogenicity and mutagenic toxicity [184,185].

Polymeric micelles, formed from amphiphilic block copolymers, are commonly used to solubilize hydrophobic drugs [186]. Despite their tunable release profiles, they often exhibit low drug loading efficiencies [187] and instability under physiological conditions, leading to premature disassembly and reduced therapeutic efficacy.

### 4.2. Therapeutic Versatility and Barrier-Penetrating Capabilities of Nano/Microgels (NG/MGs)

In contrast, nano/microgels (NG/MGs) offer a versatile and robust platform that addresses many of the limitations faced by traditional nanocarriers. Their high water content enables efficient encapsulation of hydrophilic biomacromolecules, such as proteins, DNA, and RNA, while their internal hydrophobic domains allow for the loading of hydrophobic drugs [188,189,190,191]. This dual-loading capability is particularly advantageous for combination therapies.

NG/MGs also exhibit stimulus-responsive behavior, enabling site-specific and controlled drug release in response to environmental cues such as pH, temperature, and redox conditions [21,192]. This responsiveness enhances therapeutic precision and minimizes systemic toxicity [193,194].

The effectiveness of NGs in drug delivery is often challenged by physiological barriers, which vary depending on the route of administration [195,196]. However, NG/MGs can be engineered to overcome physiological barriers associated with different administration routes. For instance, intravenously administered NGs must evade clearance by the kidneys, liver, and spleen to reach target tissues. Additionally, they may need to interact with or traverse biological membranes, and in some cases, cross the blood–brain barrier (BBB) to access the central nervous system (CNS) [197]. Mucosal delivery routes—such as oral, ocular, and pulmonary—require NGs to exhibit mucoadhesive or mucopenetrating properties to overcome the protective mucus layer covering epithelial surfaces [197]. In transdermal applications, NGs must penetrate the stratum corneum or release their payload effectively into the skin [20]. Several reviews have comprehensively discussed these physiological barriers and the strategies NG/MGs employ to overcome them [20,197]. Here, we highlight several representative examples demonstrating the potential of NG/MGs in overcoming such challenges.

#### 4.2.1. Crossing the Blood–Brain Barrier

The BBB, composed of tightly joined endothelial cells, restricts the entry of most molecules into the CNS, posing a significant obstacle for treating neurological disorders [20]. However, NGs can exploit endogenous transport mechanisms such as absorptive, cell-mediated, or receptor-mediated transcytosis. Surface modifications that target specific receptors on BBB endothelial cells further enhance their ability to cross this barrier [20].

**Figure 6:**
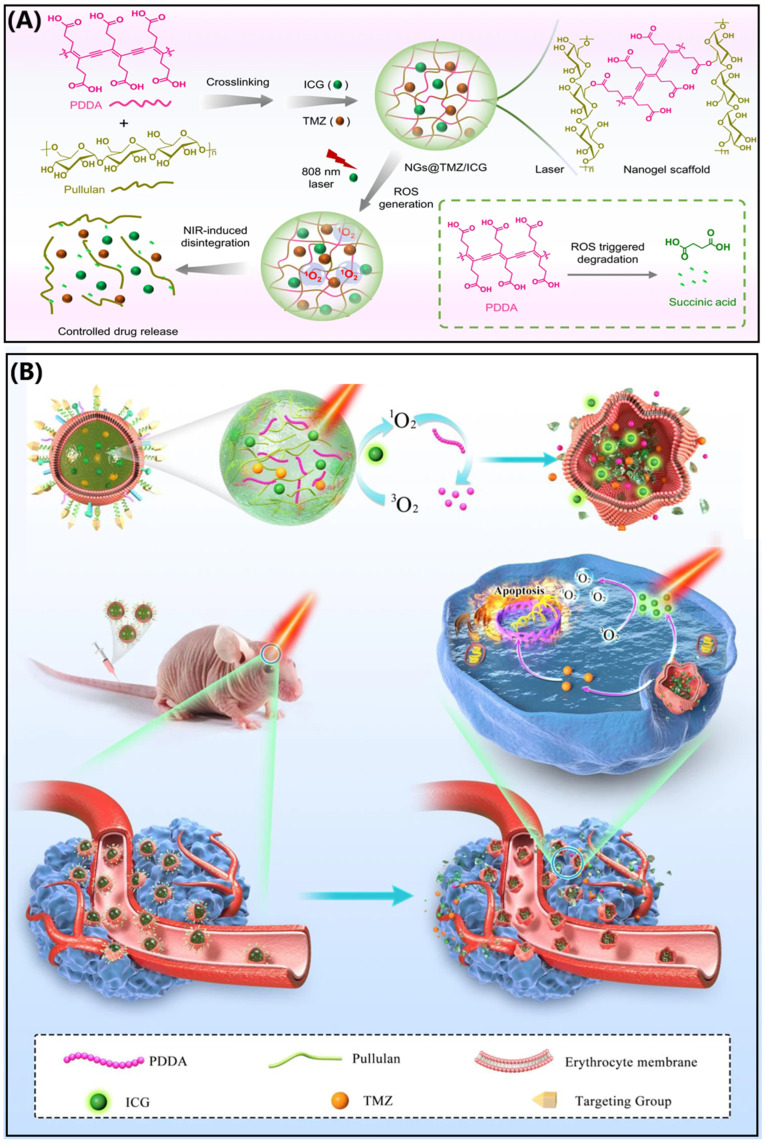
Schematic of (**A**) NG disintegration under NIR light and (**B**) NIR-activatable biomimetic NGs for glioblastoma therapy. Reproduced with permission from Zhang et al. [198], Nature Communications 2022.

A notable example is the development of a near-infrared (NIR) responsive biomimetic NG system, ARNGs@TMZ/ICG, designed by Zhang et al. for glioblastoma multiforme (GBM) therapy [198]. This system was synthesized by crosslinking poly(deca-4,6-diynedioic acid) with pullulan, followed by encapsulation of indocyanine green (ICG) and temozolomide (TMZ). The NG core was then coated with an erythrocyte membrane functionalized with apolipoprotein E (ApoE) peptide. The ApoE modification facilitated BBB penetration and tumor cell uptake via interactions with low-density lipoprotein receptors, which are overexpressed in both U87MG brain tumor cells and BBB endothelial cells. Upon NIR irradiation, ICG generated reactive oxygen species (ROS), triggering the release of TMZ specifically within GBM lesions. This targeted delivery system significantly inhibited tumor progression in both orthotopic GBM and GBM stem cell-bearing mouse models. To address the challenge of delivering drugs across the blood–brain barrier (BBB), a specialized NG system was developed using a crosslinked network of pullulan and an oxidatively degradable polymer known as poly(deca-4,6-diynedioic acid) (PDDA) (Figure 6). This polymer remains stable under normal physiological conditions but breaks down in the presence of reactive oxygen species (ROS), which can be generated by light-activated photosensitizers.

The NGs were loaded with two key agents: indocyanine green (ICG), a near-infrared (NIR) photosensitizer approved by the FDA, and temozolomide (TMZ), a standard chemotherapy drug for glioblastoma multiforme (GBM). This dual-loaded formulation, referred to as NGs@TMZ/ICG, was further coated with erythrocyte membranes modified with apolipoprotein E (ApoE) peptides. The ApoE decoration enhances circulation time in the bloodstream and facilitates targeted delivery to brain tumors by interacting with receptors overexpressed on both BBB endothelial cells and GBM cells. Once the NGs accumulate in the tumor site following intravenous administration, NIR light is applied externally to activate the system. The light triggers ICG to produce ROS, which causes the NG structure to degrade and release both TMZ and ICG. This controlled release mechanism promotes deeper penetration into the tumor tissue and enhances the therapeutic effect through a combination of photodynamic therapy and chemotherapy. In preclinical models, this NIR-responsive NG system demonstrated significant tumor suppression in mice with orthotopic GBM, highlighting its potential as a promising strategy for targeted brain cancer treatment.

#### 4.2.2. Ophthalmic Drug Delivery

Ophthalmic disorders such as cataracts, infections, glaucoma, and dry eye syndrome are increasingly prevalent [199]. Although topical formulations like eye drops and ointments are commonly used, their effectiveness is limited by rapid tear turnover, blinking reflexes, and poor corneal permeability [200,201]. NGs offer a promising alternative by enabling sustained drug release, protecting drugs from enzymatic degradation, and maintaining optical clarity and comfort due to their high water content and colloidal stability [200].

**Figure 7:**
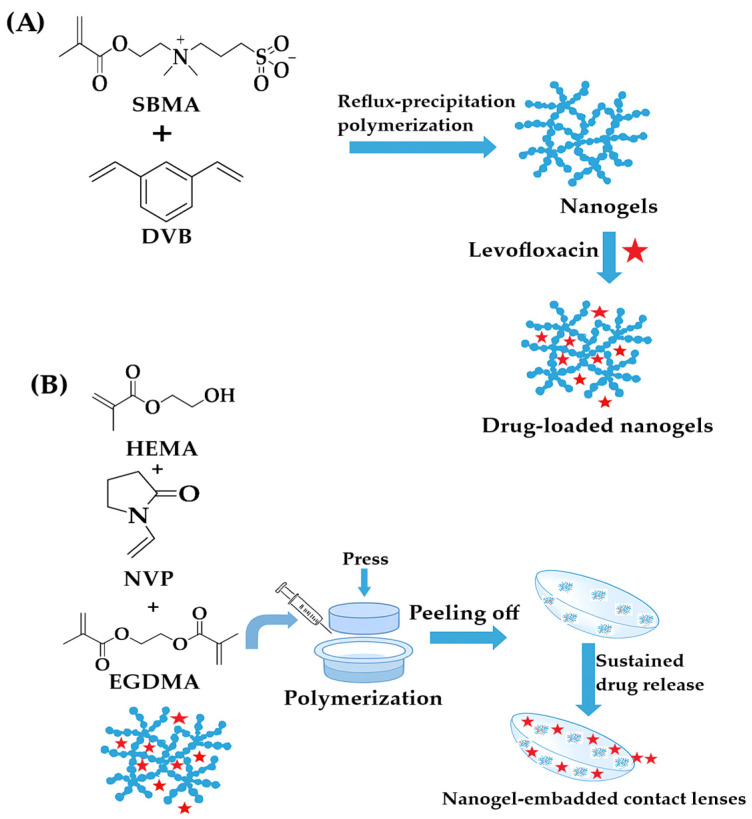
(**A**) Reflux-precipitation polymerization of NGs and preparation of drug-loaded PSBMA NGs; (**B**) Fabrication of NG-embedded contact lenses, redrawn and adapted with permission from ref. [200]. MDPI 2021.

An innovative approach was demonstrated by Wang et al., who developed therapeutic contact lenses embedded with NGs for sustained ophthalmic drug delivery [200]. They synthesized PSBMA-based NGs via one-step reflux-precipitation polymerization and loaded them with levofloxacin (LEV). These NGs were incorporated into hydrogel lenses using HEMA and N-vinyl-2-pyrrolidone monomer solutions through cast molding. The resulting lenses, containing 8 wt% NGs, exhibited excellent transparency, water retention, and cytocompatibility. Importantly, they provided sustained LEV release over 10 days without an initial burst, highlighting their potential as a comfortable and effective ophthalmic drug delivery platform (Figure 7).

NG/MGs, with their tunable synthesis, surface modification capabilities, and diverse administration routes, offer a robust platform for overcoming physiological barriers in drug delivery. Their successful application across various disease models underscores their potential in advancing therapeutic strategies for complex conditions.

#### 4.2.3. Cancer Therapy

##### Delivery of Small Molecules for Cancer Therapy Using NGs

Chemotherapy has long been a cornerstone of cancer treatment, and more recently, phototherapy has emerged as a complementary approach. Both strategies rely heavily on small molecules—chemotherapeutic agents like doxorubicin (DOX) and paclitaxel (PTX) [22,202,203,204,205,206,207,208], and photosensitizers or photothermal agents for light-based therapies [209,210]. Additionally, small-molecule inhibitors and agonists are increasingly used in cancer immunotherapy [211]. Despite their therapeutic potential, these drugs often suffer from poor bioavailability and severe side effects when administered in free form.

To address these limitations, NG-based drug delivery systems have been developed to enhance drug efficacy and reduce toxicity [211]. NGs can be administered intravenously, provided they exhibit sufficient stability, compatibility with blood components, and resistance to protein adsorption to avoid rapid clearance by the reticuloendothelial system (RES) [22,212,213,214,215].

**Figure 8:**
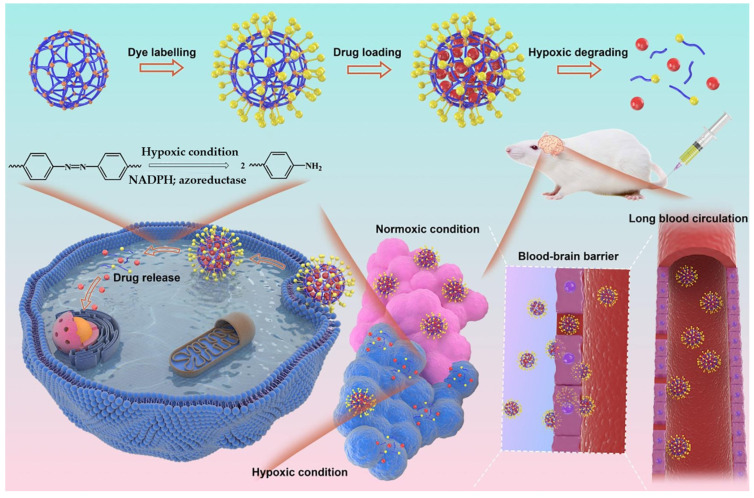
Schematic of poly(phosphorylcholine)-based (HPMPC) NG with extended circulation, BBB penetration, and hypoxia-responsive drug release for glioblastoma therapy, reproduced with permission from ref. [22]. Elsevier 2021.

A notable example is the hypoxia-responsive NG developed by She et al., known as HPMPC NG [22] (Figure 8). This system utilizes a zwitterionic phosphorylcholine polymer crosslinked with an azobenzene-based linker that degrades under low-oxygen conditions typical of tumor environments. Upon degradation, the NG releases DOX directly into the tumor. Its membrane-mimicking structure also enables it to cross the blood–brain barrier, allowing for effective accumulation and treatment of glioblastoma, a highly aggressive brain tumor.

**Figure 9:**
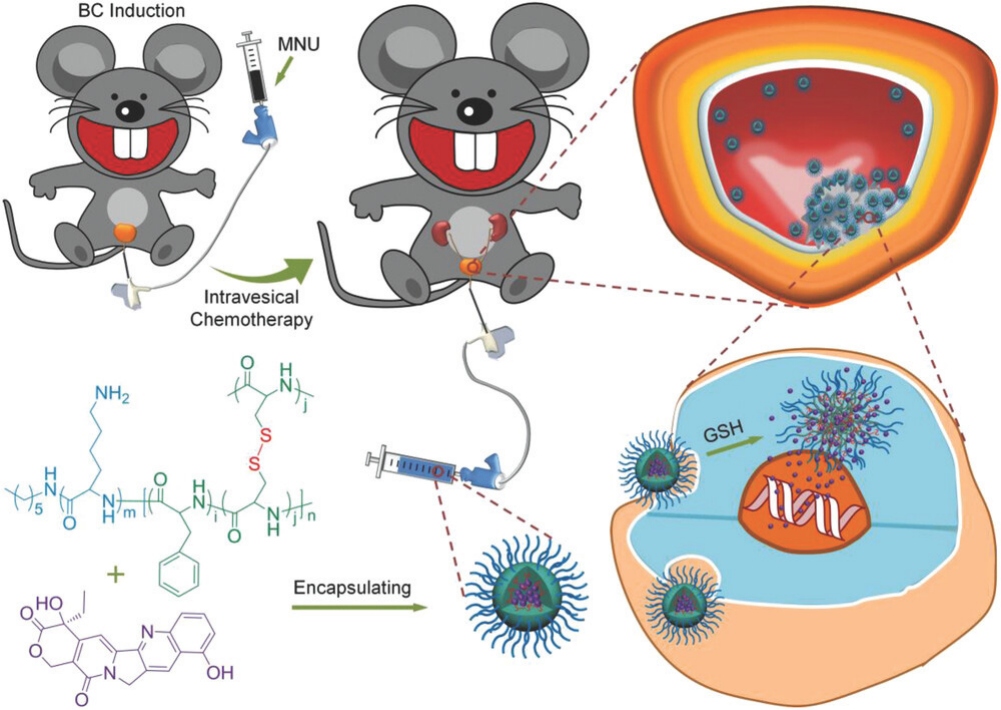
Chemical structure of NG/HCPT and its metabolic pathway in vivo. NG/HCPT accumulates in tumor tissue due to mucoadhesion and cellular uptake, with GSH-triggered HCPT release, reproduced with permission from ref. [218]. Wiley 2018.

Beyond chemotherapy, NGs are also used to deliver photosensitizers and photothermal agents via intravenous routes [216,217]. For cancers located in mucosal tissues, such as bladder [218,219] and cervical cancer [220], NGs with mucoadhesive properties [221] are particularly beneficial. Their high viscosity allows them to adhere to mucosal surfaces, increasing local drug concentration and enhancing penetration through the mucus barrier [222]. For instance, a polypeptide-based NG (NG/HCPT) was developed using poly(L-lysine)–poly(L-phenylalanine-co-L-cystine) (PLL–P(LP-co-LC)) to deliver 10-hydroxycamptothecin (HCPT) [218] (Figure 9). The cationic nature of the polymer provided strong mucoadhesion and facilitated drug penetration. Additionally, disulfide crosslinking endowed the NG with reduction-sensitive release, enabling targeted drug delivery and enhanced antitumor activity both in vitro and in vivo.

**Figure 10:**
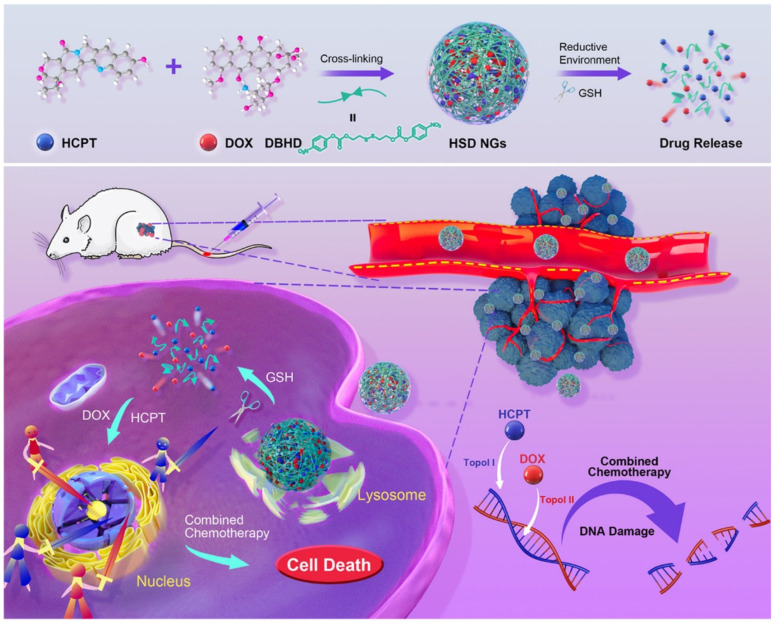
Reduction-responsive chemo-capsule-based prodrug NG for enhanced chemotherapy, reproduced with permission from ref. [202]. ACS 2021.

Given the complexity and heterogeneity of tumors, single-drug therapies often fall short, leading to incomplete tumor eradication and recurrence [219]. To overcome this, NGs have been designed to co-deliver multiple drugs with complementary mechanisms, creating a synergistic “drug cocktail” effect [223,224]. For example, Lu et al. developed a reduction-sensitive NG capable of simultaneously delivering DOX and HCPT [202] (Figure 10). The drugs were conjugated to a disulfide-containing crosslinker, allowing for controlled release in the reductive tumor microenvironment. This dual delivery approach enhanced DNA damage and improved therapeutic outcomes compared to monotherapy.

In summary, NGs offer a highly adaptable platform for small-molecule delivery in cancer therapy. Their ability to improve drug stability, target specific tissues, and enable combination treatments makes them a promising tool for advancing cancer care.

##### Delivery of Biomacromolecules for Cancer Therapy Using NGs

Beyond small-molecule drugs, NGs have also been employed to deliver biomacromolecules—such as proteins and nucleic acids—for cancer treatment. These macromolecular therapeutics often offer greater specificity and reduced toxicity compared to traditional chemotherapeutics [202].

**Figure 11:**
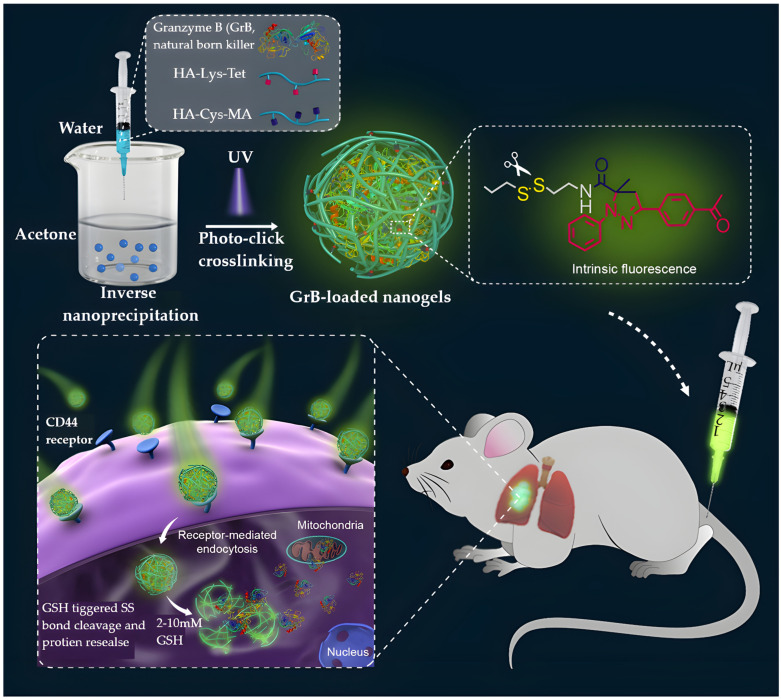
Bioresponsive fluorescent HA NGs for protein encapsulation and tumor-targeted delivery via inverse nanoprecipitation and photoclick chemistry, reproduced with permission from ref. [226]. ACS 2016.

Protein drugs can act either outside or inside cells. While extracellular proteins are more commonly used clinically, intracellular proteins require delivery systems capable of crossing cell membranes, protecting the proteins from enzymatic degradation, and releasing them in the correct cellular compartment [225]. NGs, with their high water content, excellent biocompatibility, and efficient protein-loading capacity, are well-suited for this purpose [226,227,228,229,230,231,232,233,234]. For example, Chen et al. developed hyaluronic acid (HA)-based NGs to deliver two intracellular proteins: cytochrome c (CC), which induces apoptosis [226], and granzyme B (GrB), which activates or deactivates intracellular proteins to promote cell death. Encapsulation in NGs significantly enhanced the therapeutic efficacy of both proteins, with CC showing over a tenfold improvement in potency [226] (Figure 11).

**Figure 12:**
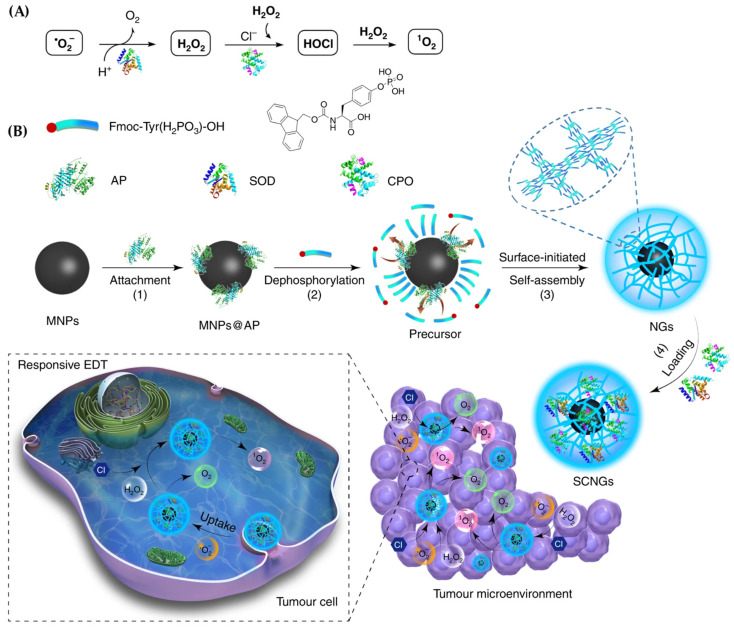
Illustration of the responsive enzyme-driven therapy (EDT) mechanism and synthesis of SCNGs; (**A**) The therapeutic cascade involves superoxide dismutase (SOD) converting superoxide radicals (˙O_2_^−^) into hydrogen peroxide (H_2_O_2_), followed by chloroperoxidase (CPO) transforming both the generated and naturally occurring H_2_O_2_ into singlet oxygen (^1^O_2_), a potent reactive species; (**B**) The SCNGs are synthesized through a multi-step process: (1) functionalization of magnetic nanoparticles (MNPs) and attachment of an alkaline phosphatase (AP) trigger, (2) AP-mediated conversion of hydrophilic peptide precursors into hydrophobic gelators via dephosphorylation, (3) AP-induced self-assembly of hydrogelators around MNPs through π–π stacking and electrostatic interactions, and (4) incorporation of SOD and CPO enzymes to enable targeted and efficient EDT within the tumor microenvironment. These responsive SCNGs facilitate the transformation of endogenous reactive oxygen species (˙O_2_^−^ and H_2_O_2_) into cytotoxic ^1^O_2_ via the SOD/CPO cascade, leading to selective cancer cell destruction. Reproduced with permission from ref. [229]. Nature Communications 2019.

Another innovative system involved neutrophil-inspired NGs carrying two enzymes—superoxide dismutase (SOD) and chloroperoxidase (CPO)—to elevate reactive oxygen species (ROS) levels inside cancer cells [229] (Figure 12). These core–shell NGs (SCNGs) were synthesized via enzyme-mediated peptide precursor dephosphorylation. The enzymes catalyzed a cascade reaction that converted superoxide into hydrogen peroxide, which was then transformed into hypochlorous acid and singlet oxygen (^·^O_2_), effectively killing cancer cells without harming healthy tissue.

**Figure 13:**
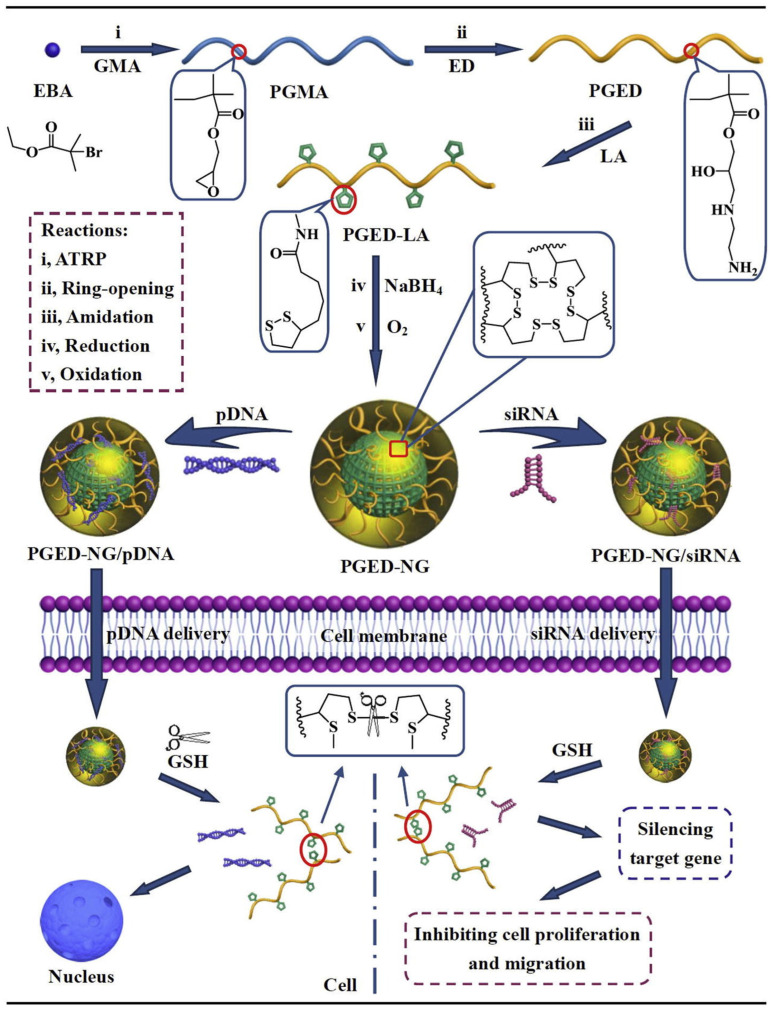
Preparation of PGED-NG and its complexes with pDNA and siRNA for nucleic acid delivery [242], reproduced with permission from ref. [201]. Elsevier 2016.

NGs have also been widely explored for gene therapy, delivering various nucleic acids such as plasmid DNA (pDNA) [229], siRNA [235,236], nucleoside analogues [237], and microRNAs (miRNAs) [238]. These molecules can regulate gene expression, silence oncogenes, or modulate immune responses [239,240,241]. For instance, NG composed of α-lipoic acid (LA) crosslinked with ethylenediamine (ED)-modified poly(glycidyl methacrylate) (PGMA) was developed for pDNA delivery [242] (Figure 13). Inside cells, the hydrophobic LA groups were reduced to hydrophilic forms, promoting pDNA release and resulting in high cellular uptake and transfection efficiency.

**Figure 14:**
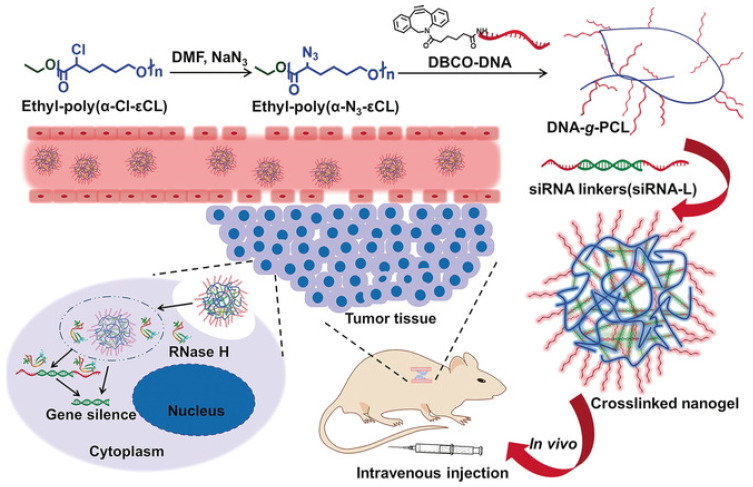
Crosslinked NG formation and siRNA delivery in vivo, reproduced with permission from ref. [243]. Wiley 2018.

In another example, siRNAs were incorporated into NGs by hybridizing with brush-like DNA-grafted polymers, forming the crosslinked structure of the gel [242]. Ding et al. used this method to deliver anti-PLK1 siRNAs, targeting the oncogene polo-like kinase 1 (PLK1) [243] (Figure 14). The NGs protected the siRNAs from degradation and enhanced cellular uptake, leading to effective gene silencing both in vitro and in vivo.

**Figure 15:**
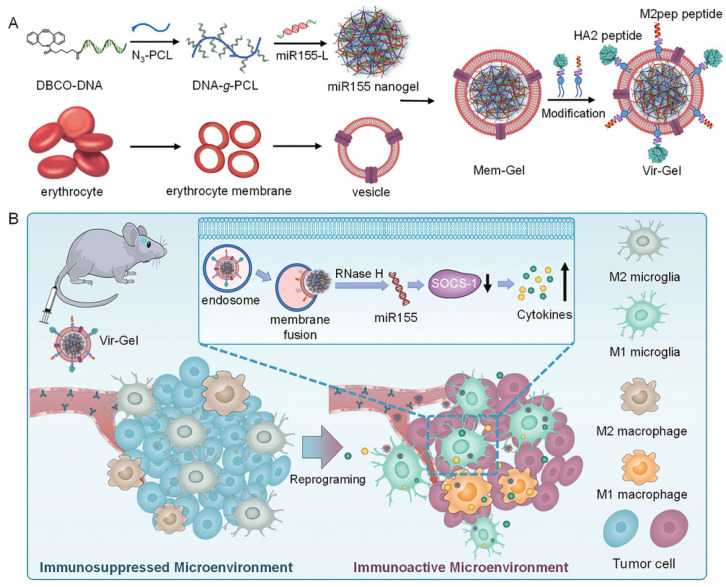
Virus-mimicking nucleic acid NG (Vir-Gel) for glioblastoma therapy. (**A**) Construction of peptide-modified, erythrocyte membrane-coated Vir-Gel; (**B**) miR155-mediated immune modulation, reproduced with permission from ref. [238]. Wiley 2021.

Additionally, miRNA-loaded NGs have been designed to modulate immune responses. Gao et al. created a virus-mimicking NG system called Vir-Gel, which encapsulated miR-155 to reprogram tumor-associated microglia and macrophages from a pro-tumor (M2) to an anti-tumor (M1) phenotype [238] (Figure 15). The NG core was coated with a red blood cell membrane and functionalized with targeting peptides to enhance cellular uptake and mimic viral infection, resulting in prolonged circulation and strong tumor inhibition.

NGs offer a powerful and flexible platform for delivering biomacromolecules in cancer therapy. Their ability to protect sensitive molecules, target specific cells, and respond to intracellular conditions makes them ideal for advancing protein- and gene-based treatments. As research progresses, the diversity of nucleic acids and proteins that can be delivered using NGs continues to expand, broadening their therapeutic potential.

##### NGs as Multifunctional Platforms for Cancer Therapy

**Figure 16:**
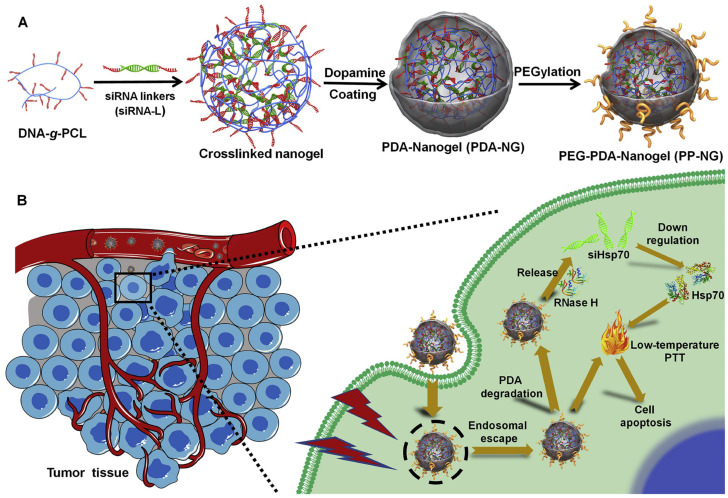
Synthesis of PDA-coated nucleic acid NG and its role in siRNA-mediated low-temperature photothermal therapy. (**A**) PEG-PDA-NG synthesis; (**B**) Mechanism of siRNA-induced PTT, reproduced with permission from ref. [257]. Elsevier 2020.

Cancer remains one of the most challenging diseases due to its complexity, heterogeneity, and resistance to treatment. To improve therapeutic outcomes while minimizing side effects, researchers have developed NG platforms capable of delivering multiple therapeutic agents or combining different treatment modalities. These platforms can carry not only small molecules and biomacromolecules but also nanoparticles, enabling a wide range of combination therapies [244,245]. NGs can enhance anticancer efficacy by: (i) Co-delivering multiple drugs to boost the effectiveness of a single treatment approach [246,247,248,249], (ii) Integrating different therapeutic strategies—such as chemotherapy, photothermal therapy (PTT), photodynamic therapy (PDT), gene therapy, and magnetic therapy—into one system [234,239,240,241,242,243,244,245,246,247,248,249,250,251,252,253,254,255,256]. For instance, Ding et al. developed an NG system that co-delivered polydopamine (a photothermal agent) and siRNAs targeting heat shock protein 70 (Hsp70) [257] (Figure 16). Traditional PTT requires temperatures above 50 °C, which can cause inflammation and promote metastasis [258,259,260,261,262,263,264]. By silencing Hsp70, which helps cancer cells resist heat-induced damage [265], the NG enabled effective tumor ablation at milder temperatures (42–45 °C), reducing side effects while maintaining therapeutic efficacy.

**Figure 17:**
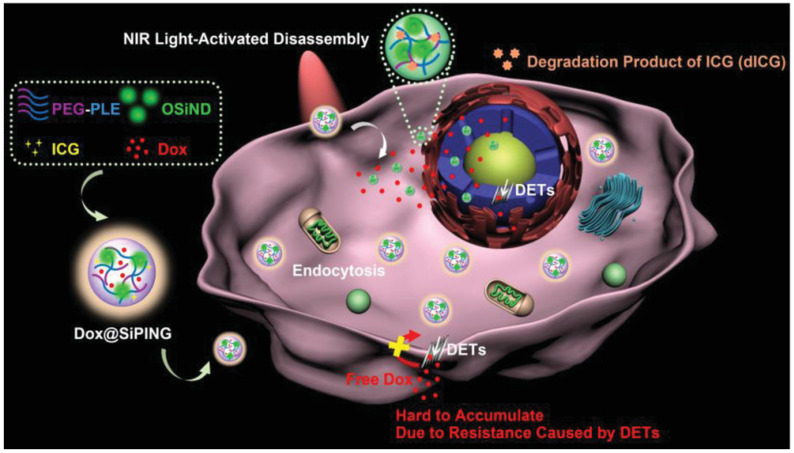
Rational design of Dox-encapsulated NGs acting as ‘Trojan horses’ for enhanced cellular uptake and nuclear drug delivery to overcome MDR, reproduced with permission from ref. [254]. Wiley 2019.

Multidrug resistance in cancer is often driven by factors like drug efflux transporters (DETs) and anti-apoptotic proteins [266,267]. To address this, a smart NG named SiPING was developed using a one-step self-assembly method. It incorporated PEG–PLE, organosilica nanodots (OSiND), indocyanine green (ICG), and doxorubicin (DOX) [254] (Figure 17). This system acted like a “Trojan horse,” helping DOX evade DETs and enter cancer cells efficiently. Upon NIR light exposure, ICG degraded, triggering the release of DOX, which rapidly reached the nucleus—even in resistant cells—resulting in strong anticancer effects.

**Figure 18:**
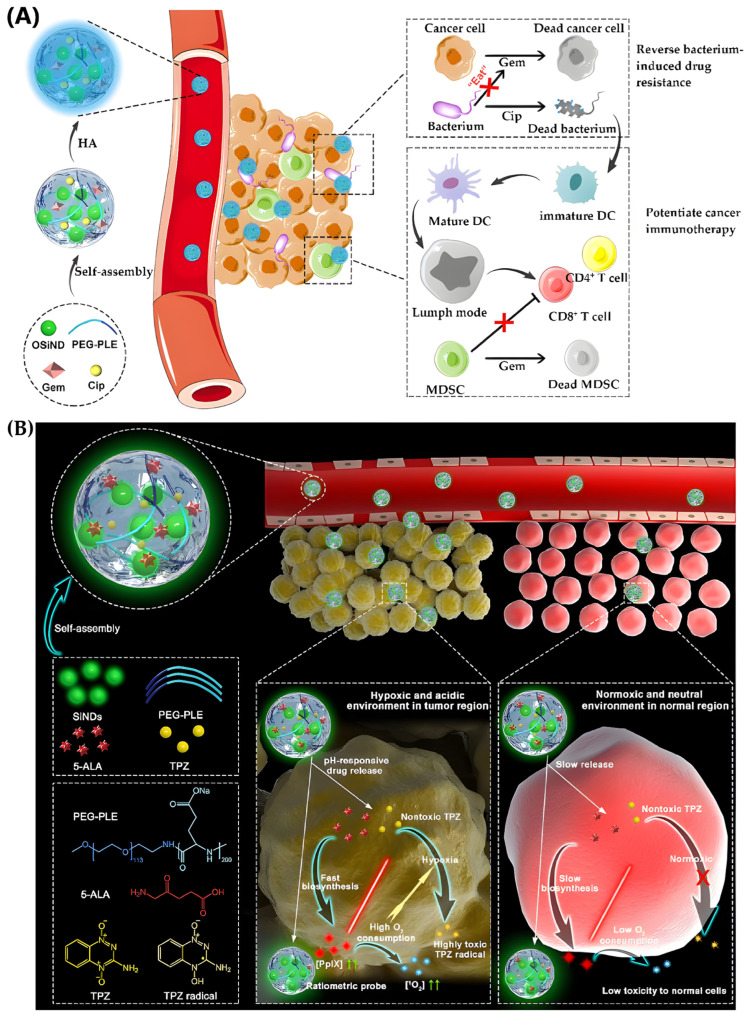
NGs for cancer therapy. (**A**) Synthesis and mechanism of Gem/Cip@SiPNG@HA nanoreservoir for immunotherapy, reproduced with permission from ref. [258]. Wiley 2021; (**B**) SiPNGs as theranostic platforms, reproduced with permission from ref. [190]. Wiley 2019.

In another study, a self-traceable nanoreservoir called Gem/Cip@SiPNG@HA was designed to treat bacterium-associated tumors [258] (Figure 18A). It combined gemcitabine (Gem), ciprofloxacin (Cip), and hyaluronic acid (HA) for targeted delivery. The system featured enzyme- and pH-responsive gates for controlled drug release in the tumor microenvironment. Since bacteria in tumors can deactivate Gem and cause resistance [258], co-delivery with Cip allowed simultaneous tumor inhibition and bacterial eradication. Moreover, the dead bacteria acted as immune stimulants, enhancing T cell-mediated immune responses and promoting antigen-presenting cell maturation, while Gem helped deplete immunosuppressive cells—demonstrating a novel strategy to turn tumor-associated bacteria into immunoadjuvants.

Some NGs are designed not only for therapy but also for diagnosis, making them powerful theranostic platforms. These systems can simultaneously deliver therapeutic agents and enable imaging or monitoring of disease progression [190,268,269,270,271,272,273,274]. In addition, NGs can support imaging-guided therapy, allowing real-time tracking of drug delivery and treatment response [235,275,276]. A notable example is the self-traceable NG SiPNG, developed by incorporating silicon nanodots (SiNDs), PEG–PLE, 5-aminolevulinic acid (5-ALA), and tirapazamine (TPZ) [190] (Figure 18B). SiNDs emit green fluorescence, enabling visualization of the NG. Once delivered to the tumor site, 5-ALA is converted into protoporphyrin IX (PpIX), a red-fluorescent molecule that serves both as a diagnostic probe and a photosensitizer. Upon light activation, PpIX generates singlet oxygen (^1^O_2_), consuming local oxygen and creating a hypoxic environment that enhances the cytotoxicity of TPZ. This dual-action system successfully combined imaging and therapy for precise cancer treatment.

**Figure 19:**
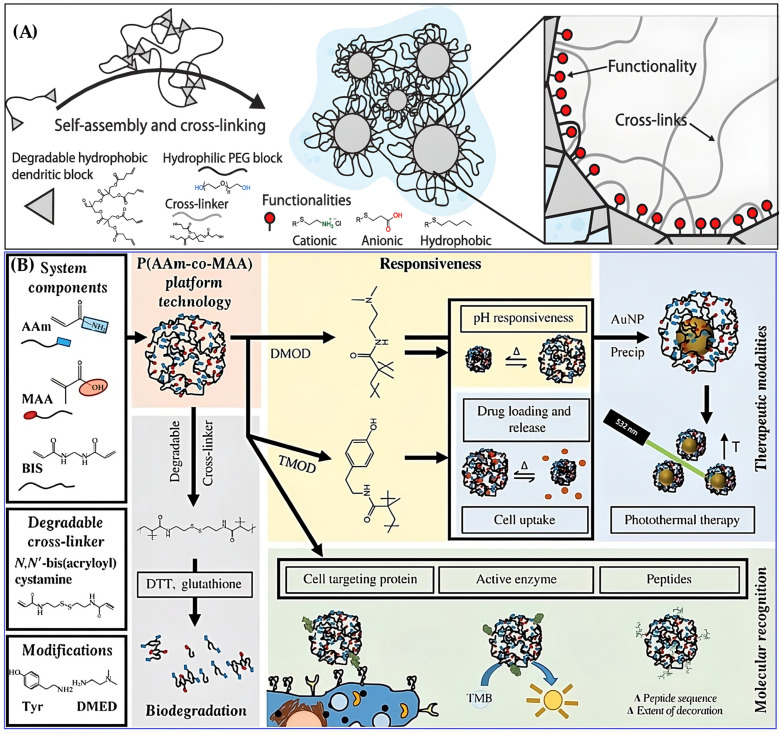
NGs for cancer therapy. (**A**) Structure and tunability of DNGs for personalized therapy, reproduced with permission from ref. [191]. Wiley 2019; (**B**) Design of P(AAm-co-MAA) NG platform for precision medicine, reproduced with permission from ref. [278]. AAAS 2019.

Beyond theranostics, NGs can be engineered for precision medicine, where their structure and surface chemistry are tailored to match specific therapeutic needs. Their ability to encapsulate a wide range of materials—regardless of size, charge, or hydrophobicity—makes them ideal for customized drug delivery [191,277,278]. For instance, the Malkoch group developed dendritic NGs (DNGs) using amphiphilic block copolymers made from hydrophilic PEG and hydrophobic hyperbranched bis-MPA dendrons [191] (Figure 19A). These were functionalized with allyl groups to allow crosslinking via thiol–ene chemistry. The resulting NGs could be further modified with various thiol-containing molecules to introduce carboxyl, amine, or alkyl groups, enabling the delivery of anionic, cationic, or hydrophobic drugs, respectively. These DNGs showed controlled drug release, low toxicity to healthy cells, and efficient uptake by 3D pancreatic tumor models.

Another example comes from the Peppas group, who created a tunable NG platform using poly(acrylamide-co-methacrylic acid) [P(AAm-co-MAA)] (Figure 19B) [278]. This system allowed: (i) Crosslinking with degradable or non-degradable linkers, enabling environment-responsive breakdown, (ii) Surface modification with small molecules or peptides, enhancing drug loading, targeting, and cellular uptake, (iii) Integration with gold nanoparticles (AuNPs) for photothermal therapy, using the NG’s reductive properties to precipitate AuNPs and enable heat generation under laser irradiation.

These features allowed the NGs to deliver multiple drugs, respond to tumor-specific stimuli, and support combination therapies—all essential for personalized cancer treatment. NGs are emerging as multifunctional platforms that bridge therapy, diagnostics, and precision medicine. Their tunable chemistry, responsiveness to biological environments, and compatibility with diverse therapeutic agents make them ideal candidates for next-generation cancer treatment strategies.

##### NGs as Cancer Vaccines and Synthetic Antibodies

Vaccination is a well-established method for preventing and treating diseases by stimulating the immune system with antigens derived from pathogens or their subunits [222,279]. While traditionally used against infectious diseases, vaccines are now being explored for cancer therapy [280] and other chronic conditions like metabolic syndrome [281].

**Figure 20:**
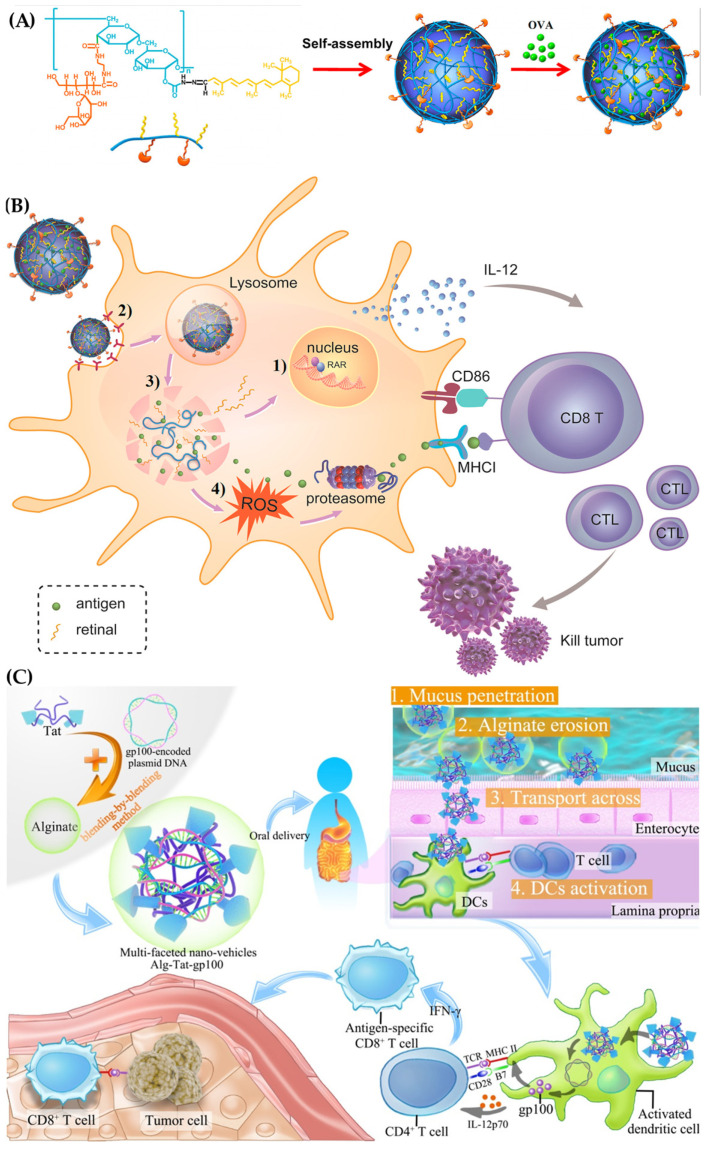
NGs as vaccines. (**A**) Synthesis of GDR NG and GDR/OVA nanovaccine; reproduced with permission from ref. [288]. (**B**) Mechanism of immune response enhancement; (**C**) Alg-Tat-gp100 NG for oral vaccine delivery, Elsevier 2016; reproduced with permission from ref. [241]. Elsevier 2019.

In the immune response, extracellular antigens are processed by antigen-presenting cells (APCs) and presented via MHC class II molecules to activate CD4^+^ T cells, leading to humoral and cellular immunity [281]. However, effective cancer vaccines require activation of CD8^+^ cytotoxic T cells (CTLs), which involves antigen presentation through MHC class I molecules [281]. Recent advances have introduced nanoparticle-based vaccines, or nanovaccines, which offer improved delivery and immune activation [282,283]. NGs, in particular, are ideal carriers due to their ability to protect antigens from degradation, enhance uptake by APCs, enable controlled release, promote antigen presentation, and stimulate APC maturation [268,273]. NG-based cancer vaccines typically encapsulate tumor-associated antigens (TAAs), which alone may not elicit strong immune responses [284]. These are often formulated as subunit vaccines [285] or derived from tumor cell lysates. For example, carboxyl-modified cholesterol-bearing pullulan (CHP) NGs showed selective interaction with APCs and strong CTL activation due to efficient cross-presentation [279]. Targeting dendritic cells (DCs) is a key strategy in NG vaccine design, as DCs are central to T cell activation [286,287,288]. Wang et al. developed galactosyl dextran-retinol (GDR) NGs loaded with ovalbumin (OVA) as a model antigen [288] (Figure 20A,B). The galactosyl groups enabled DC targeting, while retinoic acid receptor (RAR) signaling promoted DC maturation. Additionally, the NGs induced lysosomal rupture, generating ROS that enhanced proteasome activity and MHC I antigen presentation, leading to robust anticancer immune responses.

NGs can also deliver nucleic acids that encode TAAs. For instance, plasmid DNA gp100, used to treat metastatic melanoma, was encapsulated in an Alg-Tat-gp100 NG composed of alginate and Tat peptide [241] (Figure 20C). Oral delivery of this system significantly increased interferon-γ secretion and CTL activation, achieving a tumor inhibition rate of 42.5%.

**Figure 21:**
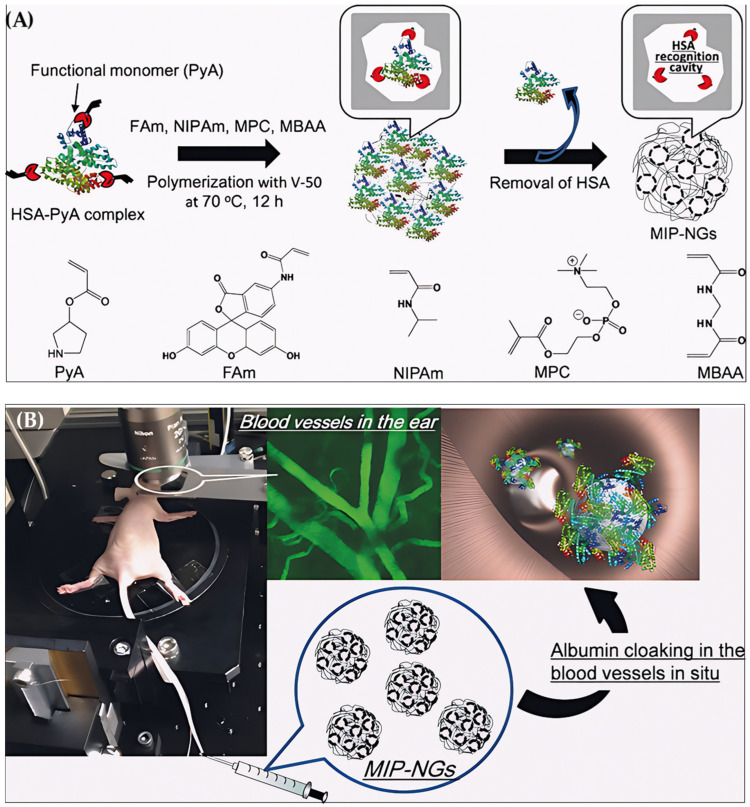
(**A**) Preparation of MIP-NGs for HSA via emulsifier-free polymerization; (**B**) Concept of stealth acquisition by albumin cloaking, reproduced with permission from ref. [291]. Wiley 2017.

Beyond vaccines, NGs can function as synthetic antibodies through molecular imprinting. These molecularly imprinted polymer (MIP) NGs are created by polymerizing around a template molecule (e.g., an antigen), which is later removed to leave specific recognition sites [289,290]. These sites allow the NGs to selectively bind target proteins, mimicking antibody behavior [291]. MIP NGs have been successfully applied in protein recognition and are being explored as antibody alternatives in cancer therapy [291] (Figure 21A,B).

NGs offer a versatile platform for cancer immunotherapy, functioning as both vaccines and synthetic antibodies. Their ability to deliver antigens or genetic material, target immune cells, and stimulate robust immune responses makes them promising tools for next-generation cancer treatments.

##### NGs for Anti-Inflammatory Therapy

NGs have emerged as promising carriers for delivering a wide range of anti-inflammatory agents, including antibiotics [292,293], nucleic acids [26], photosensitizers [26], and even bioactive polymers or metal ions [26]. Interestingly, some NGs themselves exhibit inherent anti-inflammatory properties [23,294], partly due to their hydrophilic nature, which contributes to immunomodulation [295]. In general, the more hydrophilic the NG, the stronger its ability to reduce immune responses.

Many inflammatory diseases are linked to immune system dysfunction. For example: inflammatory bowel disease (IBD) [295]. Rheumatoid arthritis (RA) [296,297] and spinal cord injury (SCI) [298,299]. To address these conditions, NGs have been developed for oral or intravenous delivery. For instance, a PEG–PEI-based NG loaded with rolipram, an anti-inflammatory drug, was designed for targeted release in SCI [300,301]. This system, called NG_Roli, effectively suppressed inflammatory markers like iNOS and Lcn2 in A1 astrocytes, leading to improved neuronal survival in treated mice.

**Figure 22:**
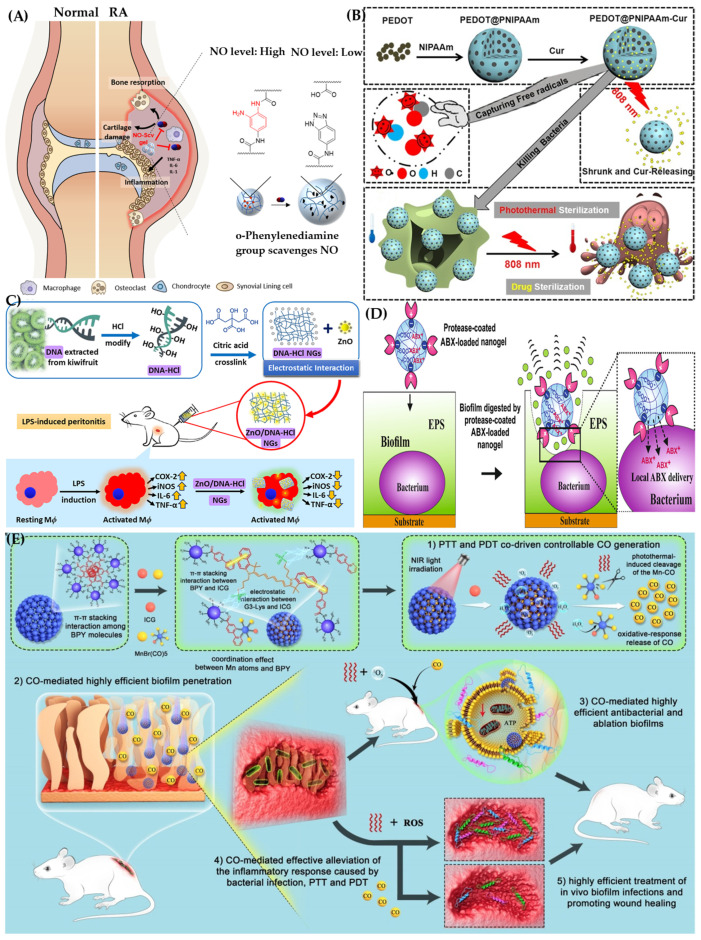
NGs for anti-inflammatory therapy. (**A**) NO-scavenging and RA treatment, reproduced with permission from ref. [302]. ACS 2019; (**B**) PEDOT@PNIPAAm-Cur NG for sterilization, reproduced with permission from ref. [306]. Wiley 2017. (**C**) DNA-HCl-ZnO NG for peritonitis; reproduced with permission from ref. [307]. Elsevier 2021; (**D**) Polyacrylic acid NGs for biofilm eradication, reproduced with permission from ref. [293]. ACS 2019; (**E**) ICG&CO@G3KBPY NG for multimodal infection therapy, reproduced with permission from ref. [308]. Elsevier 2021.

Beyond chemotherapeutic agents, nanogels have also been employed for the delivery of nucleic acids to help mitigate inflammatory responses. For instance, Yavvari et al. developed an NG for oral delivery of PIAS1-encoded DNA to treat IBD [26]. The NG protected the DNA from degradation in the harsh gastrointestinal environment and successfully suppressed NF-κB and STAT1 signaling, reducing inflammation in a mouse colitis model. Some NGs are designed to neutralize inflammatory molecules directly. Yeo et al. created an NO-scavenging NG (NO-Scv gel) using an NO-cleavable crosslinker [302] (Figure 22A). In conditions like RA [303], IBD [304], and sepsis [305], excess NO contributes to inflammation. Upon encountering NO, the NG degraded and consumed it, outperforming dexamethasone in reducing inflammation with lower toxicity in an RA mouse model.

NGs are also effective against bacterial-induced inflammation. They can deliver antibiotics and enhance their antimicrobial activity [24,309,310,311], or possess intrinsic antibacterial properties [312]. For example, berberine-loaded polyacrylic acid NGs modified with cationic polyelectrolytes adhered to bacterial surfaces, increasing drug concentration and improving antibacterial efficacy against *E. coli* and *C. reinhardtii* [311]. Another approach involves photothermal therapy (PTT). NG composed of PEDOT and curcumin (Cur) within a PNIPAAm network was activated by NIR laser to release Cur and kill bacteria while scavenging free radicals [306] (Figure 22B). This system effectively eliminated *S. aureus* and *E. coli* and showed strong antioxidant performance.

Besides antibiotics and photothermal agents, NGs can incorporate metal ions like Zn^2+^ [306] and Cu^2+^ [110], or cationic polymers such as PEI [309], guanidine-conjugated polymers [313], and antimicrobial polypeptides [314]. For example, Taxus baccata fruit-like polypeptide NGs (PNGs) made from poly(arginine-r-valine)-mannose and Zn^2+^ selectively interacted with bacterial membranes, causing membrane disruption and bacterial lysis [314]. NGs can also mimic neutrophil extracellular traps (NETs) to combat systemic infections like sepsis. Chen et al. developed a DNA-HCl-ZnO NG using kiwifruit genomic DNA crosslinked with citric acid and loaded with ZnO nanoparticles [307] (Figure 22C). This system reduced inflammatory markers (iNOS, COX2), lowered cytokine levels (IL-6, TNF-α), and decreased bacterial load in septic mice, ultimately improving survival rates.

Biofilms represent a protective mode of bacterial growth, where bacteria are embedded in a matrix of extracellular polymeric substances (EPS), including polysaccharides, proteins, lipids, and nucleic acids [315]. This matrix shields bacteria from antibiotics and immune responses, making infections harder to treat. For example, *Pseudomonas aeruginosa* in biofilm form can be up to 1000 times more resistant to antibiotics [316]. Once established, biofilms are difficult to eliminate and often lead to chronic inflammation [292]. To address this challenge, researchers have developed NGs capable of penetrating and disrupting biofilms [293,317,318,319,320]. One example involves polyacrylic acid NGs coated with the protease Alcalase 2.4 L FG and loaded with ciprofloxacin, a cationic antibiotic [293] (Figure 22D). The protease-modified NGs acted as hydrolases and esterases, breaking down the EPS matrix and allowing the antibiotic to reach the bacteria. This approach significantly reduced biofilm mass and bacterial density, with antibiotic efficacy enhanced by nearly 1000-fold compared to free drug treatment.

Like cancer therapy, biofilm treatment can benefit from multimodal NG platforms that combine different therapeutic agents. For instance, Cai et al. developed a carbon monoxide (CO)-enhanced NG system called ICG&CO@G3KBPY, which integrates photothermal therapy (PTT), photodynamic therapy (PDT), and gas therapy [308] (Figure 22E). This NG encapsulated indocyanine green (ICG) and MnBr(CO)_5_, a CO-releasing molecule, within a peptide dendrimer-based carrier. Upon NIR light exposure, the system activated PDT and PTT, while releasing CO gas to kill bacteria, reduce inflammation, and enhance ICG penetration into biofilms. This strategy proved highly effective in treating subcutaneous abscesses and urinary catheter infections, demonstrating the potential of NGs in managing biofilm-related diseases. NGs offer a powerful solution for biofilm-associated infections, combining enzymatic degradation, targeted drug delivery, and multimodal therapy. Their ability to penetrate protective bacterial matrices and modulate inflammation makes them promising candidates for future anti-infective and anti-inflammatory applications.

##### NGs for Antidiabetic Therapy

Diabetes mellitus is a chronic metabolic disorder characterized by elevated blood glucose levels (BGLs), which significantly impacts patients’ quality of life [308]. Insulin remains a cornerstone treatment, especially for individuals with type 1 diabetes and some with type 2 diabetes, requiring daily subcutaneous injections to regulate BGLs [308].

NGs, known for their excellent protein-loading capacity and biocompatibility, have been explored as carriers for insulin delivery [321,322,323,324]. For example, Wang et al. developed hydroxyethyl methacrylate (HEMA) NGs via emulsion polymerization for oral insulin administration [323]. In vivo studies showed that these NGs enhanced intestinal absorption of insulin, maintained glucose control for up to 12 h, and significantly improved bioavailability compared to free insulin.

**Figure 23:**
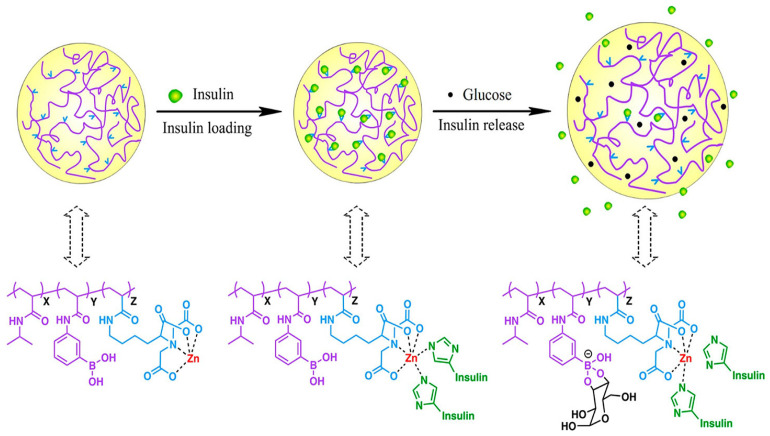
NGs for diabetes treatment. Glucose-responsive insulin release from P(NIPAM-co-AAPBA-co-AANTA) NG. Reprinted with permission from Ref. [324]. © 2018 American Chemical Society.

Traditional insulin injections cannot fully mimic the natural secretion pattern of insulin, which includes both steady release and post-meal bursts [29,322]. To address this, researchers have designed stimuli-responsive NGs that release insulin in response to glucose levels. Li et al. created a glucose-sensitive NG, P(NIPAM-co-AAPBA-co-AANTA), composed of N-isopropylacrylamide (NIPAM), (m-acrylamidophenyl)boronic acid (AAPBA), and Nε-acrylamido-Nα-bis(carboxymethyl)-lysine (AANTA). Crosslinked with ethylene glycol dimethacrylate (EGDMA), the NG was functionalized with phenylboronic acid (PBA) and nitrilotriacetic acid (NTA) to coordinate insulin via Zn^2+^ ions [324] (Figure 23). Upon glucose binding, the PBA component underwent a hydrophobic-to-hydrophilic transition, triggering insulin release in a glucose-dependent manner.

Another approach involved using glucose oxidase (GOx). Li et al. developed a dual-responsive NG containing mPEGA, AHMDM (a glucose- and H_2_O_2_-sensitive phenylboronic ester), and QT (a thiol-based crosslinker). The NG was loaded with both GOx and insulin [321]. In the presence of glucose, GOx produced H_2_O_2_, which oxidized the phenylboronic ester, causing the NG to degrade and release insulin. This system achieved controlled “on–off” insulin release, effectively reduced blood glucose levels, and maintained normoglycemia for approximately 16 h. NGs offer a promising platform for advanced insulin delivery, especially for oral administration and glucose-responsive release. Their ability to mimic physiological insulin patterns and improve bioavailability positions them as a valuable tool in the future of diabetes management.

##### NGs for Antiviral Therapy

Throughout history, viral infections have caused numerous global health crises. Notable examples include the COVID-19 pandemic caused by SARS-CoV-2 [321,325], the HIV/AIDS epidemic [326], Dengue outbreaks [327], the H1N1 influenza pandemic [328], and the emergence of the Zika virus [328]. In response to these challenges, NGs have been developed for various antiviral applications, including Antiviral drug delivery [329,330], Vaccine platforms [331,332,333], and Synthetic antibodies or antibody mimics [291,334,335]. NGs can enhance the performance of antiviral drugs by improving their stability, bioavailability, and targeting, while reducing side effects [329,330]. For example, nucleoside reverse transcriptase inhibitors (NRTIs) are widely used in HIV treatment but face challenges such as poor penetration into the central nervous system (CNS) and mitochondrial toxicity at high doses. Moreover, their active forms—NRTI 5′-triphosphates (NTPs)—are unstable in serum [330].

To overcome these limitations, Warren et al. designed a biodegradable cationic NG composed of cholesterol-modified epsilon-polylysine (CEPL) and a peptide linker that targets the ApoE receptor, facilitating CNS delivery [330]. This system, called peptide-PEG-CEPL NG, was used to encapsulate NTPs. The cationic nature of the NG neutralized the negative charge of NTPs, enhancing cellular uptake and reducing mitochondrial toxicity. The brain-targeting peptide further improved CNS accumulation. Compared to conventional NRTIs and drug cocktails, the NG/NTP formulation showed Lower neurotoxicity, Higher inhibition of HIV-1 reverse transcriptase. These results highlight the potential of NGs as efficient carriers for antiviral drugs, especially for targeting hard-to-reach tissues like the brain.

NGs are increasingly being explored as platforms for virus prevention and treatment, particularly in the development of vaccines and synthetic antibodies [336,337,338]. A successful vaccine typically requires careful selection of antigens, adjuvants, delivery systems, and scalable manufacturing strategies [339]. In this context, NGs can serve either as antigen carriers or as adjuvants, enhancing immune responses and improving delivery to immune cells. Nuhn et al. developed a degradable ketal-crosslinked NG conjugated with imidazoquinoline (IMDQ), a small-molecule TLR7/8 agonist known to stimulate strong type I interferon responses and promote Th1-type immunity [337,340]. Compared to a polymer-based IMDQ delivery system, the NG showed superior lymph node targeting and reduced systemic inflammation after subcutaneous injection [340]. The NG formulation enhanced IgG1 antibody responses and significantly lowered viral loads in the lungs of mice infected with respiratory syncytial virus (RSV), demonstrating its effectiveness as a vaccine platform.

**Figure 24:**
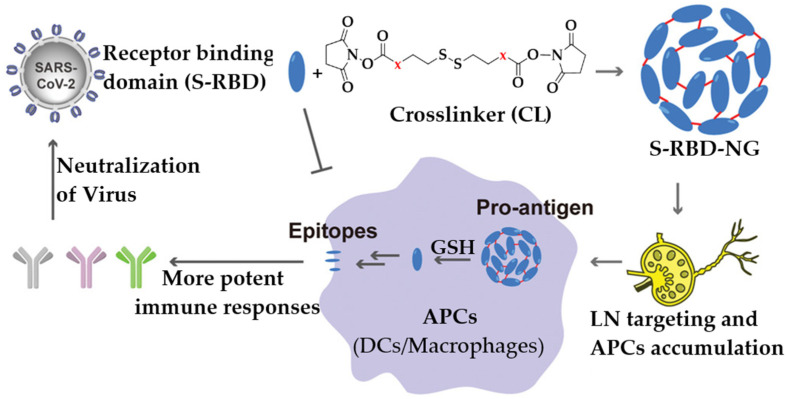
Development of a redox-responsive NG (S-RBD-NG) incorporating the SARS-CoV-2 spike receptor-binding domain (S-RBD) as a pro-antigen platform for COVID-19 subunit vaccines. The NG enhances delivery to lymph nodes and uptake by antigen-presenting cells (APCs). Once internalized, the NG gradually releases S-RBD monomers, promoting a stronger immune response capable of neutralizing the virus, reproduced with permission from ref. [338]. WILEY 2020.

In another example, Chen et al. designed a redox-responsive NG containing the receptor-binding domain (RBD) of the SARS-CoV-2 spike protein, a key antigen for COVID-19 vaccines [338] (Figure 24).

The RBD was crosslinked using disulfide-containing linkers to form S-RBD-NG, a pro-antigen nanoparticle with improved lymph node targeting and enhanced uptake by dendritic cells (DCs) and macrophages. Upon internalization, the NG degraded in response to intracellular redox conditions, releasing RBD monomers and triggering strong immune responses. This strategy demonstrated the potential of NGs in next-generation COVID-19 vaccine development.

NGs can also be engineered to mimic antibodies, either through molecular imprinting or ligand-receptor interactions. Molecularly imprinted polymer (MIP) NGs, also known as synthetic antibodies, are created by polymerizing around a target epitope, then removing the template to leave specific binding sites [291,334,335,341]. For example, Mier et al. developed a water-compatible MIP NG that blocked the binding site of Hepatitis A Virus Cell Receptor-1, showing promise in antiviral therapy [289]. In contrast, antibody mimics rely on multivalent interactions to block viral entry. Bhatia et al. synthesized a flexible sialylated NG that interacted with influenza A virus (IAV) via sialic acid residues, which bind to hemagglutinin spike proteins on the virus surface [335]. This NG effectively prevented IAV from entering MDCK-II cells and inhibited H3N2 infection, demonstrating its potential as a virus-blocking agent.

NGs offer a versatile platform for antiviral vaccines and synthetic antibody development, capable of enhancing immune responses, targeting lymph nodes, and blocking viral entry. Their adaptability and multifunctionality make them promising candidates for future virus prevention and treatment strategies.

Beyond their established roles in cancer, inflammation, diabetes, and viral infections, NGs have shown promise in a variety of other therapeutic areas, including anticoagulation, liver fibrosis, neurodegenerative diseases, immune modulation, pain relief, and even blood transfusion compatibility [342,343,344,345,346,347,348,349,350].

##### Anticoagulant and Thrombolytic Therapy

**Figure 25:**
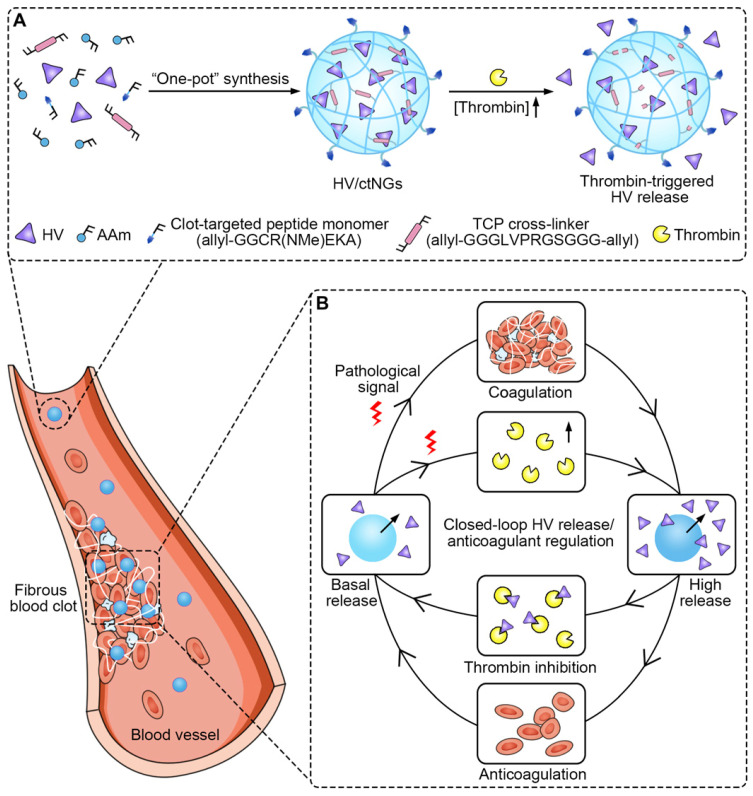
(**A**) Synthesis and thrombin-responsiveness of HV/ctNGs; (**B**) their mechanism for HV delivery and anticoagulant therapy, reproduced with permission from ref. [343]. AAAS 2020.

Blood clot formation can lead to life-threatening conditions. To address this, Xu et al. developed thrombin-responsive NGs (HV/ctNGs) for targeted delivery of recombinant hirudin variant 3 (HV), a potent thrombin inhibitor [343] (Figure 25). These NGs were synthesized using acrylamide, a thrombin-cleavable peptide crosslinker, and a clot-binding peptide. Upon encountering thrombin in clots, the NGs degraded, releasing HV to inhibit further clot formation. Notably, the system featured self-regulated release, where HV-thrombin complexes slowed further drug release, enhancing safety and prolonging circulation time. However, long-term toxicity studies are still needed.

Metabolic syndrome, which includes conditions like insulin resistance, obesity, and chronic inflammation, is a growing global health concern [351,352,353]. The gut microbiome plays a key role in its development, and recent studies suggest that NG-based immunomodulatory vaccines could help regulate immune responses and metabolic balance [281,354,355]. For example, a nasal NG vaccine containing a ghrelin–PspA fusion antigen was developed to reduce food intake and increase energy expenditure [356]. The NG enabled efficient delivery to the nasal mucosa, triggering both mucosal and systemic immune responses. In diet-induced obese mice, this vaccine led to serum IgG production and significant weight loss, highlighting its potential in obesity management.

**Figure 26:**
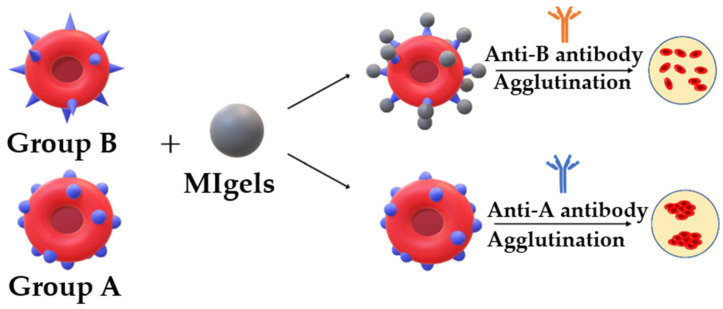
Function of MIgels in selective binding with B antigen to block antibody recognition, reproduced with permission from ref. [357]. ACS 2020.

NGs have been explored as promising tools in the context of blood transfusion. To address the challenge of blood type incompatibility, Jiang et al. created molecularly imprinted NGs (MIgels) using blood group B antigens as templates [357] (Figure 26). These MIgels selectively bound to B antigens on red blood cells (RBCs), effectively masking them and preventing agglutination during mismatched transfusions. This strategy could improve the safety and flexibility of blood transfusion practices.

NGs continue to expand their therapeutic potential across diverse medical fields. From anticoagulation and metabolic regulation to blood compatibility and neuroimmune modulation, their versatility, responsiveness, and biocompatibility make them promising candidates for next-generation biomedical applications.

## 5. Emerging Innovations and Clinical Translation in NG/MGs Technologies

The field of NG/MG technologies is rapidly evolving, driven by interdisciplinary advances in polymer chemistry, nanotechnology, biomedical engineering, and computational modeling. These soft, tunable materials are increasingly recognized as intelligent platforms capable of integrating therapeutic delivery, diagnostic imaging, and real-time physiological monitoring. As research progresses, several emerging trends are shaping the future of NG-based systems.

### 5.1. Multifunctionality and Theranostic Applications

NGs are increasingly being recognized not merely as drug carriers but as multifunctional platforms capable of integrating therapeutic delivery, diagnostic imaging, and immunomodulation [1,358,359,360]. The evolution toward “NG platforms” reflects a broader shift in biomedical engineering, where materials are designed to perform multiple roles within a single system. For example, NGs co-loaded with multiple therapeutic agents have demonstrated synergistic effects in cancer therapy [361,362], while multifunctional imaging NGs can reduce the need for repeated or multi-agent injections by enabling simultaneous visualization across modalities [363]. Theranostic NG systems are designed to diagnose, treat, and monitor disease progression simultaneously [364,365]. By incorporating diagnostic agents, therapeutic payloads, and biosensing functionalities, theranostic NGs enable real-time feedback and dynamic treatment adjustments. [9]. For example, gadolinium-loaded NGs provide MRI visibility while delivering anticancer drugs, and dual-drug systems respond to tumor-specific stimuli such as pH or enzymatic activity. These capabilities are central to personalized medicine, where therapies are tailored to individual patient profiles and adjusted based on physiological responses [366,367].

### 5.2. Immunotherapy and Vaccine Delivery

A particularly promising area is the application of NGs in immunotherapy [360]. NG-based vaccines have shown superior performance compared to free antigens or adjuvants, owing to their ability to enhance antigen stability, promote uptake by antigen-presenting cells, and facilitate controlled release. Additionally, NG-based vaccines outperform traditional formulations in both efficacy and safety. These features position NGs as strong candidates for next-generation immunotherapeutic strategies [368].

### 5.3. Hybrid Nanogels and Functional Integration

One significant development is the rise of hybrid NGs, which combine organic polymer networks with inorganic nanomaterials. This integration enhances the functional versatility of NGs, allowing them to serve as multimodal platforms for theranostics, photothermal therapy, and magnetic targeting [369,370]. Inorganic components such as gold nanoparticles enable near-infrared imaging and localized heating for cancer ablation, while silica nanoparticles improve structural integrity and drug encapsulation [371]. Iron oxide nanoparticles contribute magnetic responsiveness and MRI contrast capabilities [372]. These hybrid systems have demonstrated promising results in co-delivering therapeutic agents while simultaneously enabling imaging and targeted delivery, thereby improving treatment precision and monitoring efficiency [365]. However, their unique physicochemical properties—such as small size, high surface area, and chemical reactivity—can also pose toxicity risks, including oxidative stress, inflammation, immunogenicity, nephrotoxicity, and neurotoxicity [373]. Recent studies have identified several key factors influencing toxicity, including particle size, shape, surface charge, and colloidal stability [373]. For example, smaller NPs tend to penetrate deeper into tissues and cells, potentially causing organ-specific accumulation and cellular damage. Additionally, surface coatings and functionalization strategies are being developed to reduce off-target effects and improve biocompatibility [374]. To address these concerns, advanced omics-based approaches—including transcriptomics, proteomics, and epigenomics—are increasingly used to understand the molecular mechanisms of NP-induced toxicity. These methods help identify biomarkers and pathways affected by NP exposure, offering mechanistic insights beyond traditional cytotoxicity assays [375]. Moreover, recent material design strategies focus on biodegradable, eco-friendly, and recyclable nanohybrids, incorporating safer components and surface modifications to minimize long-term toxicity [374]. Regulatory frameworks and standardized toxicological assessments are also evolving to ensure the safe clinical translation of these materials.

### 5.4. Personalized Medicine and Smart Diagnostics

The integration of NGs into personalized medicine and smart diagnostics represents another exciting frontier [376,377]. Advances in biomarker profiling, genomic analysis, and artificial intelligence are enabling the design of NGs that respond to individual patient conditions. These smart systems can adjust drug release in response to real-time biomarker levels, integrate AI-driven diagnostic platforms to interpret sensor data, and be customized to match specific disease profiles. This convergence of nanotechnology and digital health supports the vision of precision nanomedicine, where therapies are not only targeted but also adaptive, responding dynamically to the patient’s physiological state [366].

### 5.5. Clinical Translation Challenges and Microfluidic Solutions

Despite the promising therapeutic potential of NG/MGs, their clinical translation faces several challenges, particularly in scalability, reproducibility, and long-term stability [364,365]. Traditional batch synthesis methods often suffer from variability in particle size, crosslinking density, and drug loading efficiency, which can hinder regulatory approval and large-scale production.

#### 5.5.1. Reproducibility and Regulatory Compliance

One promising solution is microfluidic synthesis, which enables precise control over reaction parameters such as flow rate, temperature, and mixing conditions. This approach allows for the continuous and reproducible fabrication of NGs with uniform size and composition [378,379]. Recent studies have demonstrated that microfluidic-assisted polymerization produces NGs with significantly narrower size distributions and enhanced batch-to-batch consistency compared to conventional emulsion techniques [33,379,380]. Moreover, microfluidic platforms can be integrated with real-time monitoring systems to ensure quality control during production [381,382].

#### 5.5.2. Scalability and Manufacturing

Scalability, once considered a limitation of microfluidic systems, is being actively addressed through parallelization and modular reactor designs, enabling production at clinically relevant volumes without compromising quality [383,384,385]. Microfluidic systems support scalable manufacturing for clinical and commercial applications. These advancements collectively contribute to overcoming key barriers in the clinical translation of NG-based therapeutics.

#### 5.5.3. Long-Term Stability

In terms of long-term stability, strategies such as lyophilization with cryoprotectants, incorporation of stabilizing agents, and surface modification with hydrophilic polymers like PEG have been shown to improve shelf-life and maintain functional integrity [386,387]. Hyaluronic acid-based MGs synthesized via microfluidic-assisted crosslinking have demonstrated sustained drug release over 30 days in vitro, with minimal structural deformation—highlighting the role of microfluidics in overcoming stability challenges [388].

## 6. Conclusions

### 6.1. Summary of Key Findings

This review has presented a detailed and structured analysis of polymer network-based NG/MGs, emphasizing their architectural diversity, synthesis techniques, and versatile applications in drug delivery and biomedical engineering. By comparing NGs/MGs, we highlighted their distinct advantages—NGs for systemic and intracellular delivery due to their small size, and MGs for localized therapies and tissue scaffolding due to their larger dimensions and mechanical tunability.

The review underscores the critical influence of polymer network parameters, such as crosslinking density and porosity, on drug encapsulation efficiency, release behavior, and mechanical properties. Stimuli-responsive systems have emerged as a transformative approach, enabling precise control over drug release and biosensing through responsiveness to environmental triggers. Functionalization strategies, including ligand conjugation, have significantly improved targeting capabilities, allowing NGs to traverse biological barriers and deliver therapeutics to challenging sites like tumors and the brain.

Importantly, the integration of hybrid NGs, multifunctional theranostic platforms, and AI-assisted diagnostics is reshaping the landscape of personalized medicine. These innovations demonstrate the growing relevance of NG/MGs in developing smart, adaptive, and patient-specific therapeutic solutions.

### 6.2. Outlook on Future Research Directions

To advance the clinical translation of NG/MG technologies, future research must focus on scalable and reproducible manufacturing methods, such as microfluidics and continuous flow synthesis, which are essential for industrial production and regulatory approval. Harmonizing regulatory frameworks will be crucial to facilitate the transition from bench to bedside.

The convergence of NGs with digital health platforms and biosensors opens new possibilities for real-time monitoring and dynamic treatment adjustment. Developing biodegradable and immunologically inert materials will enhance long-term safety and reduce adverse immune responses. Moreover, expanding the application of these systems into emerging therapeutic areas—such as regenerative medicine, immunotherapy, and neurodegenerative disease treatment—will significantly broaden their clinical impact.

In conclusion, this review not only consolidates current knowledge but also highlights the strategic importance of NG/MGs in shaping the future of precision medicine. By identifying key challenges and proposing actionable research directions, it serves as a valuable reference for researchers, clinicians, and developers working toward next-generation therapeutic platforms.

## Figures and Tables

**Table 1:** Gel Particle Architectures and Their Key Features [36].

Architecture Type	Description and Characteristics
Ultralow Crosslinked MGs	These particles have extremely low crosslinking density and swell rapidly in solvents. Despite the absence of chemical crosslinkers, physical interactions can still form a stable polymer network [122,123].
Uniform Structure	Features a consistent internal architecture with evenly distributed polymer chains and crosslinking points, resulting in homogeneous particle properties.
Core–Shell	Composed of a dense central core surrounded by a softer outer shell. This structure is typically achieved by using different monomers or crosslinkers in each region.
Core–Corona	Similar to core–shell but with a more gradual transition between core and outer regions. The crosslinking density is more evenly distributed, leading to a less defined boundary.
Hollow Particles	Created by removing the inner core through chemical or physical methods, or via degradable core strategies followed by shell formation. These are often referred to as capsules [124,125].
Multicore/Multishell	Designed to encapsulate multiple agents within separate compartments, enabling staged or sequential release of payloads [126,127,128]
Multilayered Network	Constructed through stepwise chemical or physical modifications, allowing for enhanced protection, stability, and multifunctionality within a single particle [129,130].

**Table 2:** NG/MGs Synthesis Methods [144].

	Category	Specific Technique	Description	Refs.
MGs	Homogeneous Nucleation	Emulsion Polymerization	Monomers and crosslinkers are dispersed in water to form a uniform solution. Polymerization occurs under conditions that prevent macrogel formation.	[145]
	Emulsification: W/O Heterogeneous Emulsion	Emulsion Polymerization	Water-soluble monomers and bioactives are emulsified in oil using surfactants and shear forces. Gelation is induced chemically or thermally.	[146,147]
	Inverse Microemulsion Polymerization	Inverse Emulsion Polymerization	Monomer droplets are dispersed in an oil phase by mechanical stirring, allowing for the incorporation of drugs and other substances. Crosslinking agents are used.	[148]
	Membrane Emulsification	Membrane Emulsification	An emulsion is passed through membrane pores into a nanofluid phase to create MGs.	[131]
	Heterogeneous Controlled/Living Radical Polymerization	Controlled Radical Polymerization	Includes stable radical polymerization, RAFT, and transfer radical polymerization techniques.	[131]
	Polymer Complexation	Polyelectrolyte Complexation	MGs are formed by mixing oppositely charged polymer solutions, resulting in polyelectrolyte complexes.	[67]
	Radiation	Radiation-Induced Polymerization	Polymer solution is placed in molds and exposed to γ-rays for crosslinking and sterilization. Hydroxyl radicals from water radiolysis react with polymer chains.	[149,150]
	Physical-Based Methods	Photolithographic Techniques	Monomer solution with photoinitiator and crosslinker is exposed to UV or laser light. Masks and stamps control MG size and shape.	[148]
		Micromolding Method	Polymer solution is placed in a mold, and gelation occurs via temperature change or gelling agent.	[151]
		Microfluidic and Droplet Formation	Polymer solutions are injected into an oil phase and crosslinked using microfluidic devices to form particles with specific morphologies.	[147]
NGs	Traditional Method	Emulsion Polymerization	Simultaneous polymerization and crosslinking in an emulsion system.	[137,140]
		Precipitation Polymerization	Polymerization in a solvent where the polymer is insoluble, forming NGs.	[137]
		Inverse Emulsion Polymerization	Polymerization in aqueous droplets dispersed in the oil phase.	[137]
		Dispersion Polymerization	Polymerization in a medium where monomers are soluble but polymers precipitate.	[137]
		Controlled Radical Polymerization	Includes ATRP, RAFT, and iodine-mediated polymerization techniques.	[152]
		Uncontrolled Radical Polymerization	Polymerization without control over molecular weight or structure.	[137]
	Crosslinking of Polymer Precursors	Disulfide-Based Crosslinking	Crosslinking via disulfide bonds for redox-responsive NGs.	[137,140]
		Amine-Based Crosslinking	Crosslinking via amine groups for stable NG networks.	[137,140]
		Imine Crosslinking	Formation of imine bonds for pH-sensitive NGs.	[92,105]
		Click Chemistry-Based Crosslinking	Efficient crosslinking using azide-alkyne cycloaddition.	[137,140]
		Photoinduced Crosslinking	Crosslinking is initiated by light exposure.	
		Physical Crosslinking	Non-covalent interactions are used for reversible NG formation.	
		Controlled Aggregation	Self-assembly of hydrophilic polymers into NGs.	[141]
	Template-Assisted Fabrication	Photolithography	NGs formed using light exposure through templates.	[137,141,153]
		Micromolding Techniques	NGs formed by molding polymer solutions.	[137,141,153]
	Novel Methods	Pullulan Chemistry Modification	Modified pullulan used for novel NG synthesis.	[153]
		Photochemical Approach	New photochemical techniques for NG formation.	
		Radical Polymerization with Inverse Mini-Emulsion	Combines radical polymerization with mini-emulsion technology.	
		Addition-Fragmentation Transfer Process	Advanced polymerization technique for NG synthesis.	
		Chemical Modification	Chemical alteration of polymers to form NGs.	

**Table 3:** Comparative Summary of Two NG/MGs Fabrication Routes.

Attribute	Inverse Microemulsion	Radiation-Induced
Typical size & dispersity	20–200 nm; narrow (droplet-templated) [158]	50–300 nm; dose- and concentration-dependent [12]
Encapsulation bias	Hydrophilic biomolecules (aqueous core) [157]	Broad; limited by radiation sensitivity [162]
Chemistry scope	Water-soluble vinyl monomers (AAm, NIPAM, PEG-MA) [157].	Radiation-sensitive polymers (PVA, PEG, PVP, chitosan, PAA) [161].
Process intensity	High surfactant load; batch-wise [156].	Initiator-free; sterilization built-in; continuous possible [161].
Main bottlenecks	Surfactant removal; scale-up limitations [156].	Facility access; payload degradation risk [161].
Recent highlights	Tunable pH/thermal responses; green formulations [156].	Cancer-targeted pH-responsive systems; clean injectables [162].

**Table 4:** Scalability and Application Relevance.

Feature	Inverse Microemulsion Polymerization	Radiation-Induced Polymerization
Scalability	Limited (batch-wise) [163]	High (continuous processing) [161]
Biocompatibility	Moderate (surfactant residues) [164]	High (initiator-free) [161]
Sterilization	Requires a separate step [164]	Built-in during synthesis [161]
Polymer Compatibility	Hydrophilic monomers [163]	Radiation-sensitive polymers [161]
Encapsulation of Biomolecules	High [164]	Moderate (radiation-sensitive) [161]
Industrial Feasibility	Low to moderate [163]	High [161]

## Data Availability

No new data were created or analyzed in this study.

## Abbreviations

The following abbreviations are used in this manuscript:

Abbreviation	Full Term
5-ALA	5-Aminolevulinic Acid
APC	Antigen-Presenting Cell
ATRP	Atom Transfer Radical Polymerization
ATP	Adenosine Triphosphate
AuNPs	Gold Nanoparticles
BBB	Blood–Brain Barrier
CC	Cytochrome c
CHP	Cholesterol-bearing Pullulan
CLSM	Confocal Laser Scanning Microscopy
CMC	Critical Micelle Concentration
Con A	Concanavalin A
CPO	Chloroperoxidase
CPT	Camptothecin
CS	Chitosan
CTL	Cytotoxic T Lymphocyte
DC	Dendritic Cell
Dex	Dextran
DMA	Dynamic Mechanical Analysis
DMEM	Dulbecco’s Modified Eagle Medium
DOX	Doxorubicin
DSC	Differential Scanning Calorimetry
DLS	Dynamic Light Scattering
DNA	Deoxyribonucleic Acid
ECM	Extracellular Matrix
EDX/EDS	Energy-Dispersive X-ray Spectroscopy
EMA	European Medicines Agency
EPR	Enhanced Permeability and Retention
FBS	Fetal Bovine Serum
FDA	Food and Drug Administration
FTIR	Fourier Transform Infrared Spectroscopy
GOx	Glucose Oxidase
GrB	Granzyme B
GSH	Glutathione
HA	Hyaluronic Acid
H&E	Hematoxylin and Eosin
H_2_O_2_	Hydrogen Peroxide
HPLC	High-Performance Liquid Chromatography
IC_50_	Half Maximal Inhibitory Concentration
IBD	Inflammatory Bowel Disease
ICG	Indocyanine Green
IMDQ	Imidazoquinoline
IPN	Interpenetrating Polymer Network
IVIS	In Vivo Imaging System
LCST	Lower Critical Solution Temperature
MG	Microgel
MHC	Major Histocompatibility Complex
miRNA	MicroRNA
MIP	Molecularly Imprinted Polymer
MPA	2,2-Bis(hydroxymethyl)propionic acid
MPN	Metal–Phenolic Network
MTT	3-(4,5-Dimethylthiazol-2-yl)-2,5-diphenyltetrazolium bromide
MTX	Methotrexate
MW	Molecular Weight
NETs	Neutrophil Extracellular Traps
NG	Nanogel
NIR	Near-Infrared
NIR-II	Second Near-Infrared Window
NMR	Nuclear Magnetic Resonance
NO	Nitric Oxide
NPs	Nanoparticles
NTP	Nucleoside Triphosphate
PAMAM	Poly(amidoamine)
PAAm	Polyacrylamide
PAA	Poly(acrylic acid)
PBS	Phosphate-Buffered Saline
PBA	Phenylboronic Acid
PCL	Polycaprolactone
PDT	Photodynamic Therapy
PEG	Polyethylene Glycol
PEG–PLE	Poly(ethylene glycol)-poly(L-glutamic acid)
PEI	Polyethylenimine
PHEMA	Poly(2-hydroxyethyl methacrylate)
PLGA	Poly(lactic-co-glycolic acid)
PMMA	Poly(methyl methacrylate)
PNIPAM	Poly(N-isopropylacrylamide)
PNVCL	Poly(N-vinylcaprolactam)
PpIX	Protoporphyrin IX
PspA	Pneumococcal Surface Protein A
PTT	Photothermal Therapy
PVA	Polyvinyl Alcohol
PVP	Polyvinylpyrrolidone
pDNA	Plasmid DNA
PDI	Polydispersity Index
RA	Rheumatoid Arthritis
RAFT	Reversible Addition–Fragmentation Chain Transfer
RAR	Retinoic Acid Receptor
RBD	Receptor-Binding Domain
RES	Reticuloendothelial System
RGD	Arginine-Glycine-Aspartic Acid
RNA	Ribonucleic Acid
ROS	Reactive Oxygen Species
RPM	Revolutions Per Minute
RSV	Respiratory Syncytial Virus
RT	Room Temperature
SCI	Spinal Cord Injury
SEM	Scanning Electron Microscopy
SiNDs	Silicon Nanodots
siRNA	Small Interfering RNA
SOD	Superoxide Dismutase
TAA	Tumor-Associated Antigen
TEM	Transmission Electron Microscopy
TGA	Thermogravimetric Analysis
TLR	Toll-like Receptor
TME	Tumor Microenvironment
TMZ	Temozolomide
TPZ	Tirapazamine
UV-Vis	Ultraviolet–Visible Spectroscopy
VPTT	Volume Phase Transition Temperature
XRD	X-ray Diffraction
NG/MG	Nanogel/Microgel
